# CircCNOT6L modulates alternative splicing of SLC7A11 via splicing factor SRSF2 to confer ferroptosis resistance and promote metastasis in prostate cancer

**DOI:** 10.1038/s12276-025-01540-y

**Published:** 2025-09-29

**Authors:** Ji Liu, Maskey Niraj, Xiaojun Zhu, Yadong Guo, Zhijin Zhang, Aimaitiaji Kadier, Zhuoran Gu, Hong Wang, Libin Zou, Changcheng Guo, Bin Yang, Junfeng Zhang, Shiyu Mao, Xudong Yao

**Affiliations:** 1 Department of Urology, Shanghai Tenth People’s Hospital, Tongji University School of Medicine, Shanghai, China; 2 Urologic Cancer Institute, Tongji University School of Medicine, Shanghai, China; 3 School of Medicine, Tongji University, Shanghai, China; 4 Bhaktapur International Hospital, Bhaktapur, Nepal; 5 Department of Urology Surgery, The Affiliated Hospital of Inner Mongolia Medical University, Hohhot, China

**Keywords:** Prostate cancer, Metastasis, Tumour biomarkers

## Abstract

Prostate cancer (PCa) metastasis has emerged as a leading cause of mortality globally. Owing to the distinctive looping structure, circular RNA has become an ideal biological tumor marker. Here we investigate the mechanism and function of circular RNA, specifically circCNOT6L, on PCa metastasis. A loss-of-function assay was conducted in vitro to assess the impact of circCNOT6L on cancer cell proliferation, migration, invasion and ferroptosis. In addition, a xenograft mouse model was used to elucidate the influence of circCNOT6L on subcutaneous tumor xenograft and lung metastasis. Biochemical experiments elucidated the molecular mechanism by which circCNOT6L promotes malignant progression in PCa cells by modulating ferroptosis. Moreover, the combination of circCNOT6L-si and a ferroptosis activator was tested in organoids to evaluate their potential as suppressors of tumorigenesis. The novel circular RNA, circCNOT6L, was highly expressed in both PCa metastatic tissues and cells. circCNOT6L suppression resulted in a notable inhibition in PCa cell migration, invasion and ferroptosis in vitro. Furthermore, circCNOT6L inhibition hindered the growth and metastasis of mouse xenografts. Mechanistically, circCNOT6L, generated by the RNA-binding protein EIF4A3, competes with miR-143-5p for binding, thereby facilitating SRSF2-dependent splicing of SLC7A11 precursor RNA. This process inhibited ferroptosis in PCa cells and promoted PCa progression. Finally, inhibiting circCNOT6L overexpression in combination with the ferroptosis activator (erastin) significantly suppressed the viability of PCa-derived organoids. In conclusion, in the present study, we found that circCNOT6L induced by EIF4A3 through the SRSF2–SLC7A11 axis effectively inhibits ferroptosis, which in turn promotes malignant progression of PCa.

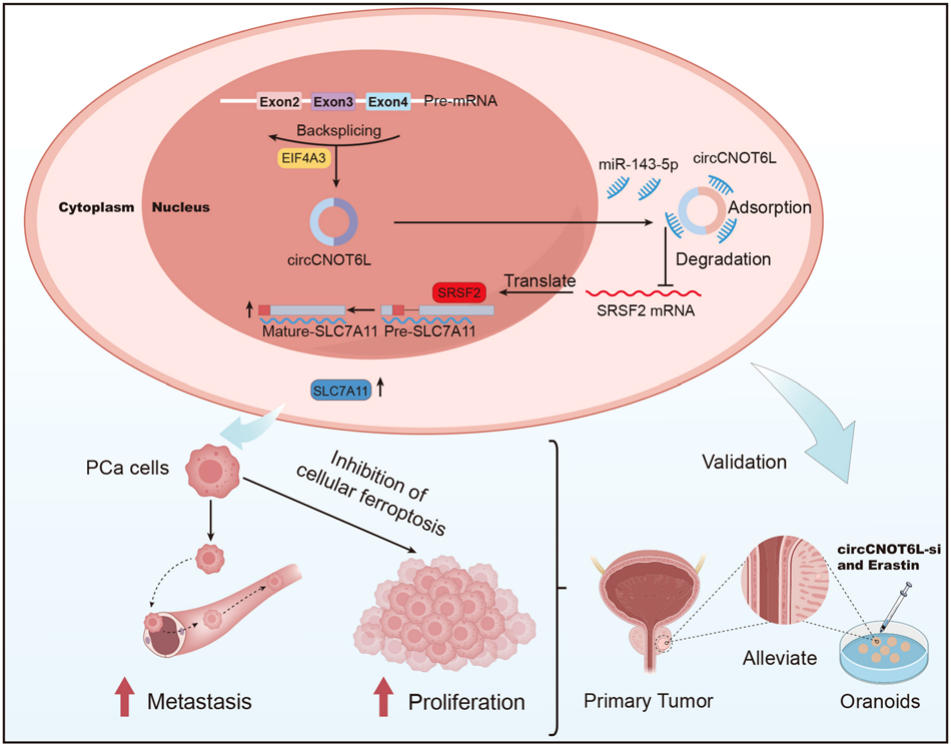

## circCNOT6L enhances prostate cancer metastasis through ferroptosis

Prostate cancer is the most common cancer in men worldwide. While treatments exist, the spread of cancer to other parts of the body makes it difficult to cure. This study explores how a molecule called circCNOT6L affects prostate cancer progression. Researchers collected tissue samples from patients and used advanced techniques to study circCNOT6L, a type of circular RNA. They found that circCNOT6L is abnormal in prostate cancer tissues and helps the cancer spread by interacting with other molecules such as SRSF2 and miR-143-5p. These interactions prevent a process called ferroptosis, which inhibits the metastasis of cancer cells. The study showed that reducing circCNOT6L levels, especially when combined with drugs that promote ferroptosis, could slow down cancer progression. This suggests that targeting circCNOT6L might be a promising new way to treat prostate cancer in the future.

## Introduction

Prostate cancer (PCa) is the most commonly diagnosed cancer in males globally, and it is the second and fifth cause of mortality in males in the USA and the world, respectively^1,2^. Despite a decrease in PCa-associated mortality, the emergence of castration-resistant PCa presents a growing challenge in disease management^3^. While radical prostatectomy remains the recommended treatment for localized PCa, metastatic spread of the tumor transforms it into an incurable condition^4^. The metastasis of PCa is the primary factor contributing to the bleak prognosis among patients with this condition^5^. Therefore, there is an imperative need to elucidate the molecular mechanisms underlying PCa metastasis and explore novel therapeutic targets for the management of PCa.

Circular RNA (circRNA), a class of noncoding RNAs with a unique closed-loop structure, plays a significant role in human diseases, including cancers^6,7^. Generated through back-splicing processes, circRNAs exhibit stable expression and resistance to exoribonuclease degradation, making them ideal biomarkers^8^. Extensive evidence has demonstrated the involvement of circRNAs in the modulation of tumor-related physiological and pathological mechanisms, including parental gene regulation, protein complex scaffolding, RNA–protein interactions and serving as microRNA (miRNA) sponges^5^.

Serine and arginine-rich splicing factor 2 (SRSF2), a widely expressed splicing factor, plays a regulatory role in transcription activation and elongation. Through its interaction with the transcription factor E2F1, SRSF2 promotes gene activities involved in cell-cycle regulation. Furthermore, SRSF2 has been shown to enhance RNA stability^9^. Notably, while SRSF2 has been implicated in the pathogenesis of various cancers, including myelodysplastic syndromes^10^, renal^11^ and gastric cancers^12^, its role in PCa remains uncertain.

Ferroptosis, an iron-dependent nonprogrammed cell death, has been associated with the progression of degenerative diseases and tumors^13,14^. Iron toxicity is characterized by the peroxidation of membrane lipids, alterations in intracellular iron levels and a decline in antioxidant defenses. The ferrometabolic response, involving changes in iron, lipid, reactive oxygen species (ROS) and glutathione (GSH) levels, indicates a close association between cellular metabolism and this form of cell death^15^. Cystine–glutamate antagonist system Xc- subunit 11 (SLC7A11) is the major transporter of extracellular cystine^16,17^. Cysteine depletion or chemicals such as erastin that block SLC7A11-mediated cystine uptake can trigger ferroptosis^14^. Ferroptosis has gained significant recognition owing to its pivotal involvement in tumor suppression^18^. Research findings have elucidated that those diverse mechanisms, including posttranscriptional regulation, epigenetic regulation and translational regulation, can modulate ferroptosis and impact cancer progression^19,20^. These findings underscore the potential of targeting ferroptosis as a novel therapeutic avenue for PCa.

This study observed a significant upregulation of circCNOT6L in PCa. Subsequent in vitro and in vivo experiments demonstrated that circCNOT6L suppresses the malignant progression of PCa by inhibiting cellular ferroptosis through the SRSF2–SLC7A11 axis. Further experiments revealed that the binding of circCNOT6L-silencing RNA (siRNA) to the ferroptosis activator effectively inhibits the proliferation of PCa cells. Overall, these findings offer a promising approach for targeting the pathogenesis and therapeutic intervention of PCa.

## Materials and methods

### Clinical specimens

Five pairs of PCa primary tumors and their metastatic lymph node tissues were collected from Shanghai Tenth People’s Hospital of Tongji University (Shanghai, China). These tissues were confirmed by the pathologist. Every study involving patients has been carried out in accordance with The Code of Ethics of the World Medical Association (Declaration of Helsinki). All the specimens were immediately frozen in liquid nitrogen after resection and later used for RNA extraction. Informed consent was obtained from the patients, and the study was approved by the Ethics Committee of Shanghai People’s Tenth Hospital (approval no. SHSY-IEC-4.1/20-22/01). In a comparable manner, a total of 25 pairs of PCa and paracancerous tissues were procured from Shanghai Tenth People’s Hospital of Tongji University. Thereafter, 20 benign prostatic hyperplasia tissues, 20 PCa tissues and 20 metastatic prostate cancer (mPCa) tissues were procured from the same hospital. Subsequently, RNA extraction and analysis were conducted using quantitative reverse transcription polymerase chain reaction (RT–qPCR) methodology. In addition, transcriptomic data and clinical information pertaining to 499 PCa cases, 35 normal tissue samples and 92 metastatic castration-resistant PCa samples were acquired from The Cancer Genome Atlas (TCGA) database. The details of the clinical samples are presented in Supplementary Table 1.

### circRNA array analysis

The patient samples were subjected to high-output transcriptome sequencing (RNA-seq) technique for analysis. Sample preparation followed Arraystar’s standard protocol, with the use of RNase R to digest linear RNA and preserve circRNAs. RNA extraction was carried out using the QubitRNA Assay kit (cat. no. Q32852, Life Technologies). The RNA-seq and data analysis were performed by OE Biotech (Shanghai OE Biotech). For bioinformatics analysis, circRNAs were identified using both find_circ and CIRI2 software. The circRNAs detected by either of these tools were combined to represent the total circRNAs identified. These circRNAs were annotated with their corresponding parent genes on the basis of their genomic locations. Known circRNAs were cross-referenced and identified using CircBase and CIRCpedia databases. Quantification of circRNAs was conducted using reads per billion, while differential expression was calculated with DEGseq. Statistical significance was determined using the negative binomial distribution test. circRNAs with a *q*-value < 0.05 and a fold change >2 or <0.5 were classified as differentially expressed. Detailed results of the differential analysis are presented in Supplementary Table 2.

### Cell culture

The Chinese Academy of Sciences supplied the PCa cell lines: PC3(CRL-1435), DU145(HTB-81), LNCaP(CRL-1740), 22RV1(CRL-2505) and normal human prostate cell line RWPE-1(CRL-3607). The cells were cultured in Roswell Park Memorial Institute-1640 medium that was cultured in defined keratinocyte serum-free medium (1×) (Gibco). RWPE-1 cells were cultured in defined keratinocyte serum-free medium (Invitrogen). The medium was supplemented with 10% fetal bovine serum (Gibco) and penicillin/streptomycin (100 μg/ml) (HyClone). All cells were cultured at 37 °C and 5% CO_2_. Details of siRNA, short hairpin RNA and overexpressed RNA are presented in Supplementary Table 5.

### Three-dimensional Matrigel drop invasion assays

This experiment was referenced from Hsu et al.^21^ A total of 1 × 10^5^ cancer cells carrying GFP protein were added to 10 μl 100% Matrigel, seeded in 24-well plates and the cell droplets were imaged using fluorescence microscopy on days 0 and 6. The medium was changed every 3 days. Cell migration out of the droplet was measured by red fluorescent protein signals using ImageJ software. The GFP fluorescence signal of the cells is indicated by the orange pseudocolor. All the experiments were performed in triplicate.

### Animal experiments

The animal care committee of Shanghai Tenth People’s Hospital of Tongji University granted approval for all animal experiments conducted (Approval no. SHDSYY-2021-3028). BABL/c male nude mice were kept under specific pathogen-free conditions. To establish a subcutaneous xenograft mouse model, a total of 12 male nude mice at the age of 5 weeks were randomly assigned to two groups. These groups were then injected subcutaneously with either 5 × 10^6^ PC3-Control or PC3-sh-circCNOT6L cells. The size of the resulting tumors was measured twice a week using vernier calipers throughout the feeding period. After a duration of 6 weeks, the mice were killed and the tumor xenografts were weighed and subjected to immunohistochemistry (IHC) analysis. In the second model, the cells were injected via the lateral tail vein of BALB/c nude mice (*n* = 6 in each group). The mice were monitored weekly, and their weight and growth were measured.

### Lipid ROS assay

C11-BODIPY 581/591 (RM02821), a lipid peroxidation sensor, was used to detect lipid reactive oxygen species (ROS) in PCa cells. For this experiment, the cells were treated for 96 h with various agents, followed by 30 min of dark incubation with C11-BODIPY probe at 37 °C. The cells were washed three times with phosphate-buffered saline before flow cytometry was used to detect C11-BODIPY (FITC, 484 nm/510 nm).

### Ferrous ion fluorescence assay

Cells were reinoculated in Petri dishes according to Ion Fluorescent Probe-Mito-Ferro Green kit instructions (Tongren) and then incubated with 5% CO_2_ at 37 °C. Following removal of the medium, the cells were washed three times with Hank’s Balanced Salt Solution buffer or serum-free medium. They were then incubated with 5% CO_2_ at 37 °C for 30 min, whereafter the supernatant was removed and the cells washed three times with Hank’s Balanced Salt Solution buffer or serum-free medium. The cells were then again incubated with 5% CO_2_ at 37°C, with medium-containing activators (for example, erastin and RSL3). The cells were photographed using confocal fluorescence microscopy thereafter.

### Organoids

Tumor tissue was obtained by radical prostatectomy and subsequently sliced into 1–2-mm pellets. These pellets were then digested for 1 h with type IV collagenase (1 mg/ml, 40510ES76, Yeason) and Y-27632 (HY-10071, MCE, 10 μM). Reconstituted pellets were seeded into prewarmed 24-well plates using Basement Membrane Extract (3533-001-02). To culture the cells, human prostate organoids were added after the Basement Membrane Extract solidified.

### Statistical analysis

Clinical data were obtained from the TCGA database to generate heatmaps and volcano maps using the heatmap package (https://cran.rproject.org). This study used the log_2_(normalized value + 1) data format for survival analysis and the R survival package for identification of progression-free interval (PFI). Statistical analysis was performed using SPSS software, version 26.0 (SPSS), and GraphPad, version 9 (GraphPad). Data are expressed as mean ± standard deviation. All experiments were performed three times, and the average of these were taken as results. *P* values <0.05 were considered statistically significant.

## Results

### CircCNOT6L is upregulated in PCa tissues and cell lines

**Fig. 1:**
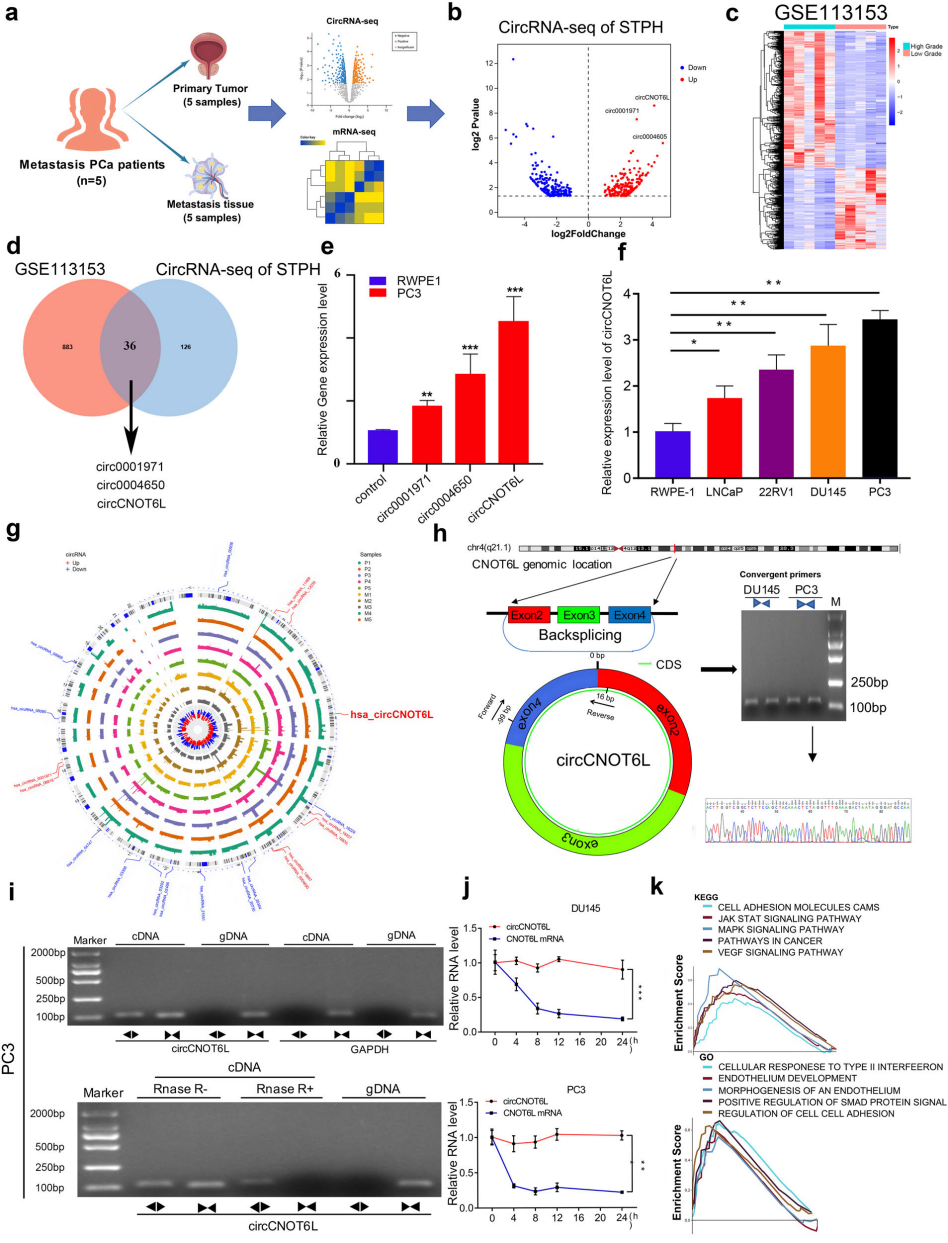
Identifying the study gene as circCNOT6L. **a** From left to right: clinical samples sequencing flow chart. **b** Volcano plot showing differential expression of circRNAs between primary and metastatic tumor tissue. **c** Heatmap showing differentially expressed circRNAs between PCa and normal tissues from GSE113153. **d** The Venn plot shows the top three circRNAs that were differentially upregulated in both GSE113153 and sequencing results. **e** RT–qPCR results showing the expression of target circRNAs in RWEP-1 versus PC3 cell lines. **f** RT–qPCR results showing circCNOT6L expression in RWEP-1, LNCap, 22RV1, DU145 and PC3 cell lines. Northern blotting showed that circCNOT6L could only be detected in cDNA and not in gDNA. **g** Circular diagram showing the location of circRNAs on chromosomes. **h** Schematic map of circCNOT6L formation and Sanger sequencing of the circCNOT6L splice junction. **i** Northern blotting assay experiments suggested that circCNOT6L was detected in cDNA but not in the gDNA group in PC3 cell lines. Furthermore, RNase R and actinomycin D treatment assays showed no change in circCNOT6L expression levels, whereas mRNA expression was significantly decreased. **j** Actinomycin D further confirms the stability of circCNOT6L. **k** GSEA shows the possible functions of circCNOT6L and the signaling pathways it is involved in. ^*^P < 0.05, ^**^P < 0.01, ^***^P < 0.01.

circRNA sequencing was conducted using five pairs of PCa tissues and their corresponding metastatic lymph node tissues (Fig. 1a). RNA-seq findings revealed 22,676 circRNAs, with 438 exhibiting significant differential expression (|fold change| >2.0 and *P* value <0.05). Among these, 208 genes were upregulated, while 230 genes were downregulated (Fig. 1b). Differential analysis was used to identify genes exhibiting differential expression between normal and paracancerous tissue samples within the GSE113153 dataset (Fig. 1c). Subsequently, three circRNAs were selected from the 36 intersecting circRNAs. We ranked these 36 circRNAs, which exhibited significantly higher expression in metastatic foci and high Gleason scores, according to the *P* values from the differential expression analysis conducted at our center and selected the top three circRNAs for further investigation (Fig. 1d). Among these, circCNOT6L was selected owing to it having the highest level of expression in PCa PC3 cell lines (Fig. 1e). Next, circCNOT6L expression levels were assessed in various PCa cell lines, with PC3 and DU145 exhibiting high and LNCaP displaying relatively low expression (Fig. 1f). Reverse and forward primers were designed from complementary DNA (cDNA) and genomic DNA (gDNA) from PCa cells for amplification of circCNOT6L and linear CNOT6L messenger RNA (mRNA), respectively. The result revealed that circCNOT6L was detected in cDNA but not in the gDNA group in DU145 cell lines (Supplementary Fig. 1a). The circular diagram illustrates the chromosomal positions of the 36 differential genes (Fig. 1g). CircCNOT6L is formed by reverse splicing of exons 2, 3 and 4 of the host gene CNOT6L, and the Sanger sequencing confirmed the back-splicing binding site of circCNOT6L (Fig. 1h). The RNase R enzyme treatment experiment showed that the expression level of circCNOT6L did not change, while that of CNOT6L mRNA decreased significantly, suggesting that circCNOT6L is a highly stable circRNA (Fig. 1i and Supplementary Fig. 1a). Actinomycin D treatment further revealed the stability of circCNOT6L over 24 h, in contrast to the significant decrease in CNOT6L mRNA expression after 4 h (Fig. 1j). These results suggest that circCNOT6L serves an oncogenic role in PCa tissues and cell lines. The results of gene set enrichment analysis (GSEA) indicated that circCNOT6L may be closely related to vascular endothelial formation and intercellular adhesion and potentially involved in the VEGF and MAPK signaling pathways (Fig. 1k). The above results suggest a potential role of circCNOT6L in PCa metastasis.

### circCNOT6L promotes the progression and metastasis of PCa cells in vivo

**Fig. 2:**
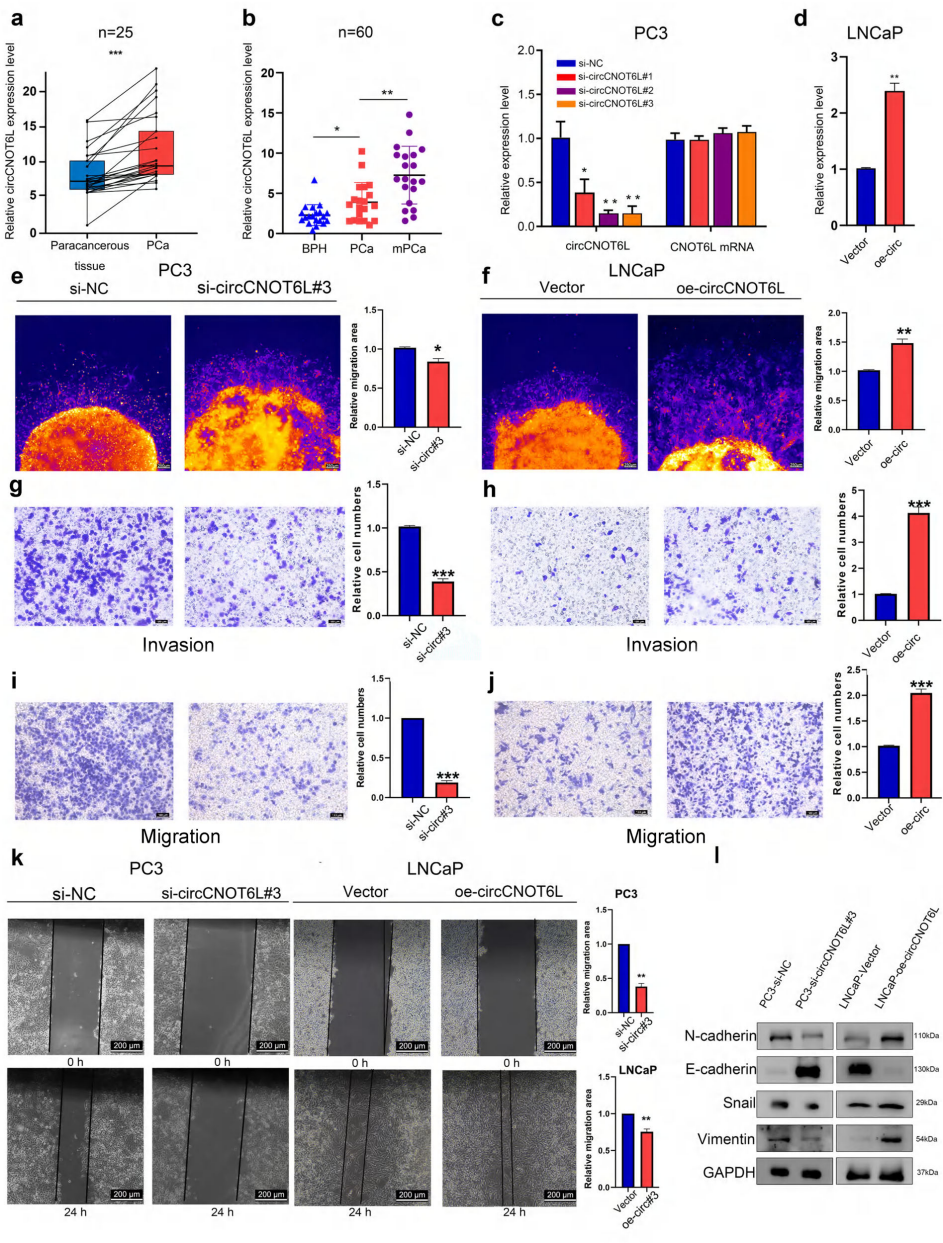
circCNOT6L facilitates PCa cell invasion and metastasis at in vivo levels. **a** RT–qPCR was used to identify the levels of circCNOT6L expression in 25 paired PCa tissues and adjacent normal tissues. **b** RT–qPCR was used to identify the expression of circCNOT6L in the urine of 60 patients with benign prostatic hyperplasia, localized PCa and metastatic PCa. **c**, **d** RT–qPCR analysis was used to determine the knockdown efficiency of si-circCNOT6L in the PC3 cell line (**c**) and the overexpression efficiency of oe-circCNOT6L in the LNCaP cell line (**d**). **e**–**h** 3D Matrigel drop invasion assay and transwell invasion assay showed the effect of circCNOT6L on the invasive function of PCa cells. **i**–**k** Transwell migration assay with wound-healing assay showed the effect of circCNOT6L on the migratory function of PCa cells. **l** Western blotting demonstrated the impact of changes in the expression levels of circCNOT6L mRNA on the expression of EMT-related proteins. ^*^P < 0.05, ^**^P < 0.01, ^***^P < 0.001.

To gain deeper insights into the role of circCNOT6L, the detection of circCNOT6L in PCa tissues and paracancerous tissue was further compared in 25 pairs of samples. The results showed that its expression was higher in PCa tissues (Fig. 2a). circCNOT6L expression was higher in mPCa, followed by PCa and then benign prostatic hyperplasia tissues (Fig. 2b). The function of circCNOT6L in PCa cells was verified by constructing siRNA against circCNOT6L. Knockdown of circCNOT6L significantly reduced the level of circCNOT6L in PC3 and DU145 cell lines, while overexpression in the LNCaP cell line was confirmed by detecting circCNOT6L expression (Fig. 2c,d and Supplementary Fig. 1b). Subsequent assays, including colony formation, CCK8 and EdU, demonstrated that circCNOT6L knockdown inhibited the proliferation of DU145 and PC3 cells compared with their negative controls (Supplementary Fig. 1c–e). Wound healing, transwell assays and three-dimensional (3D) Matrigel drop invasion assays showed that circCNOT6L knockdown inhibited the migration and invasion of PC3 and DU145 cells compared with their negative controls. Conversely, overexpression of circCNOT6L promoted the migration and invasion of LNCaP cells compared with their negative controls (Fig. 2e–k and Supplementary Fig. 1f,g). Western blotting was performed to detect the effect of circCNOT6L on the expression of epithelial–mesenchymal transition (EMT) markers. The results showed that circCNOT6L knockdown upregulated E-cadherin expression and downregulated N-cadherin, vimentin and snail expression. By contrast, circCNOT6L overexpression led to the downregulation of E-cadherin and the upregulation of other EMT markers (Fig. 2l).

### circCNOT6L promotes the progression and metastasis of PCa in vitro

**Fig. 3:**
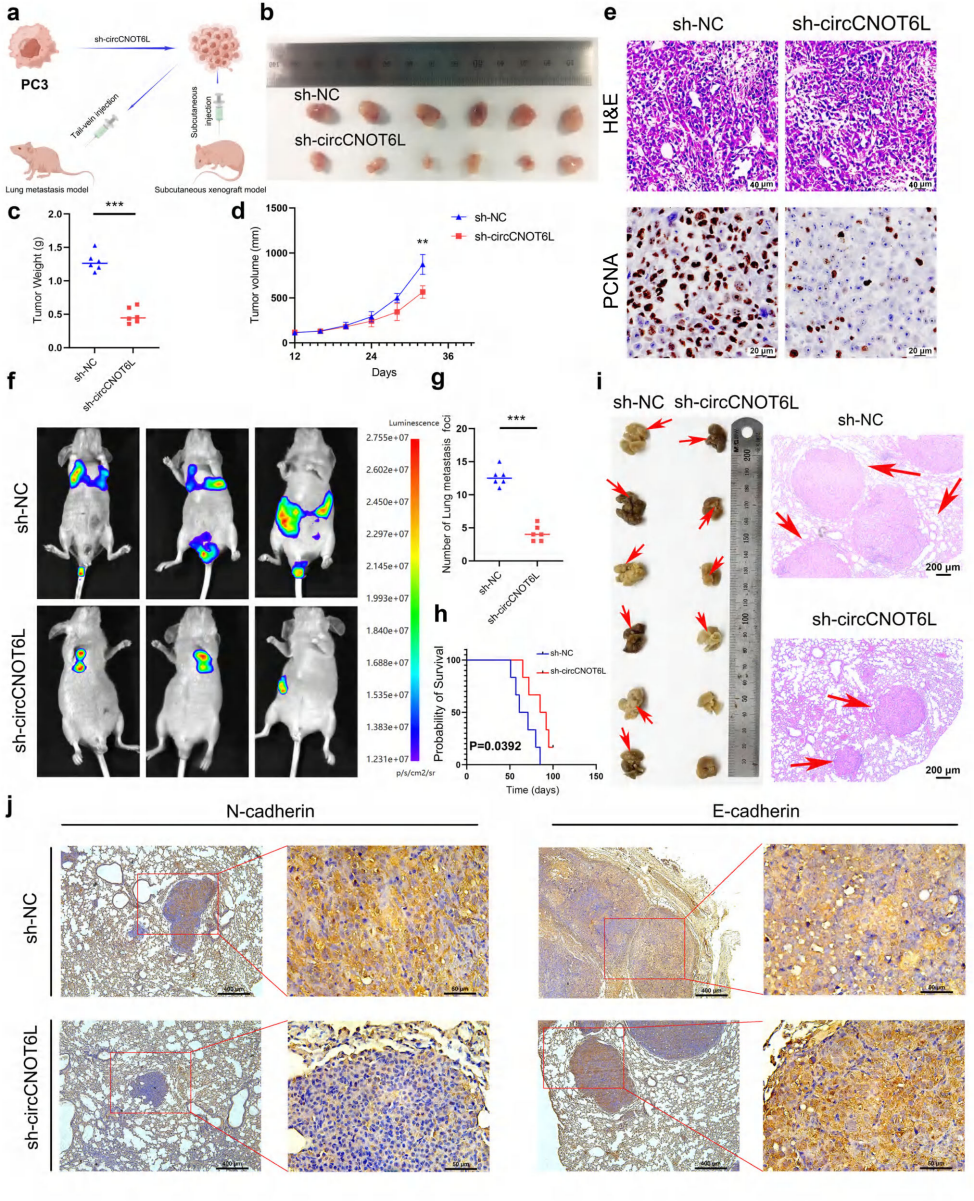
circCNOT6L promotes malignant progression of PCa cells at in vivo levels. **a** Illustration of a subcutaneous tumor and lung metastasis model in nude mice. Nude mice were injected with PC3 cells of sh-NC and sh-circCNOT6L subcutaneously and through the tail vein, respectively. **b**–**e** Tumor samples derived from subcutaneous xenograft mice underwent IHC analysis (**b**) and tumor weight (**c**) and growth (**d**) were assessed (**e**). **f**–**h** The animal live imaging system was used to characterize the growth of lung tumors in nude mice (**f**) and to assess the number of metastatic foci of the tumor (**g**) in relation to the survival curve of the nude mice (**h**). **i**, **j** H&E staining reveals the size of the tumors in various samples (**i**), while IHC staining demonstrates the correlation between the expression of circCNOT6L and EMT-related proteins. (**j**) ^*^P < 0.05, ^**^P < 0.01, ^***^P < 0.001.

Stable transfectants of sh-circCNOT6L were constructed, and the knockdown efficiency was determined (Supplementary Fig. 1h). Subsequently, the nude-mouse subcutaneous xenograft tumor model and lung metastasis model were established by injecting target cells into the subcutaneous tissue and tail vein of nude mice, respectively (Fig. 3a). Results from the subcutaneous xenograft tumor model showed that circCNOT6L knockdown significantly suppressed the volume and weight of subcutaneous tumors in nude mice (Fig. 3b–d). The IHC result demonstrated a positive correlation between the expression of PCNA (a cell proliferation marker) and circCNOT6L (Fig. 3e and Supplementary Fig. 3m). In the mouse lung metastasis model, the sh-circCNOT6L group exhibited significantly fewer metastatic foci in the lungs of nude mice, and the prognosis was significantly improved compared with that of the normal control (NC) group (Fig. 3f–h). The results of hematoxylin and eosin staining (H&E) suggested that the volume of metastatic foci in the lungs of nude mice in the sh-circCNOT6L group was significantly smaller than that of the NC group (Fig. 3i). The IHC results suggested a positive correlation between N-cadherin expression and circCNOT6L, while E-cadherin expression was negatively correlated with circCNOT6L (Fig. 3j). These findings underscore the influence of circCNOT6L on the proliferation and metastatic ability of PCa cells in vivo.

### CircCNOT6L regulates SRSF2 by competitive adsorption of miR-143-5p and promotes malignant progression of PCa

**Fig. 4:**
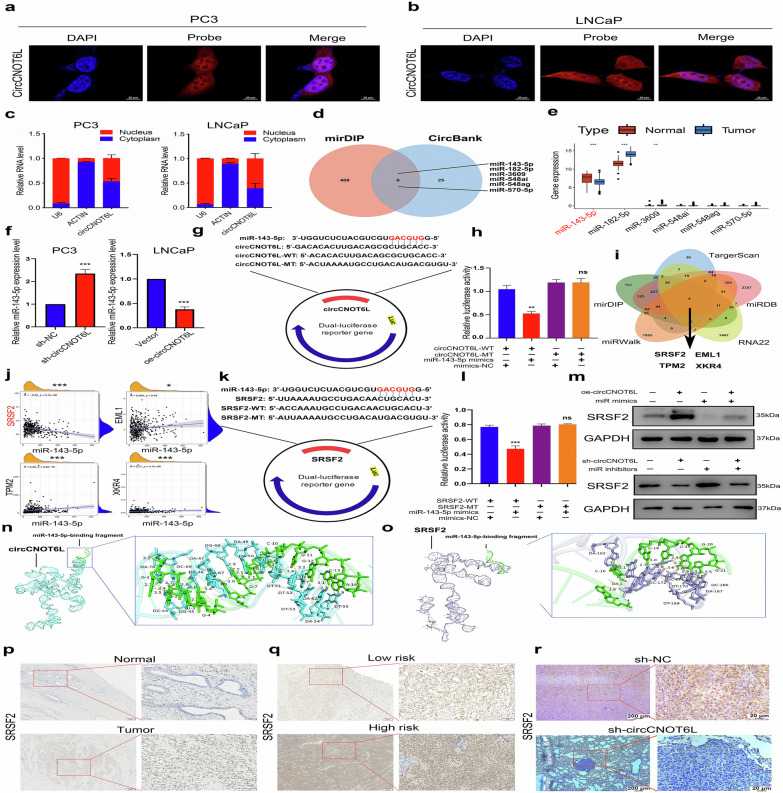
CircCNOT6L regulates SRSF2 by competitive adsorption of miR-143-5p and promotes malignant progression of PCa. **a–c** Intracellular distribution of circCNOT6L was detected by RNA fluorescence in situ hybridization and nuclear-plasma extraction assay. **d** Wayne plots showing downstream miRNAs predicted by mirDIP in conjunction with the CircBank database. **e** Box plots showing differential expression of six miRNAs in normal versus tumor samples. **f** RT–qPCR showing changes in miR-143-5p expression after altering circCNOT6L expression in PC3 and LNcap cell lines. **g**, **h** Dual luciferase reporter gene plasmid designed to detect binding of circCNOT6L to miR-143-5p. **i** Wayne plot showing miRDB, miRWalk, Targetscan and RNA22 combined with mRNAs predicted downstream by the TGCA database. **j** Co expression analysis based on the TCGA-PRAD cohort showed correlation of the screened genes with miR-143-5p. **k**, **l** Dual luciferase reporter gene plasmid designed to detect binding of SRSF2 to miR-143-5p. **m** Western blot demonstrating the correlation between circCNOT6L, miR-143-5p and SRSF2. **n** Three-dimensional structure prediction demonstrates the binding pattern of circCNOT6L to miR-143-5p. **o** Three-dimensional structure prediction demonstrates the binding pattern of miR-143-5p to SRSF2. **p**, **q** IHC results from patients with PCa demonstrated differential expression of SRSF2 between normal and tumor sample (**p**) and demonstrated differential expression of SRSF2 in tissues from patients with high-risk (Gleason score of >7) and low-risk (Gleason score of <7) samples (**q**). **r** The IHC results of lung metastasis in nude mice demonstrate a correlation between circCNOT6L and SRSF2. ^*^P < 0.05, ^**^P < 0.01, ^***^P < 0.001.

To elucidate the molecular mechanisms by which circCNOT6L promotes the malignant progression of PCa, we used fluorescence in situ hybridization and nucleoplasmic isolation experiments and investigated the localization of circCNOT6L within PCa cells. We found a predominant cytoplasmic localization of circCNOT6L in the PCa cell line (PC3/LNCaP) (Fig. 4a–c). This cytoplasmic distribution suggested a potential role for circCNOT6L as a molecular sponge, competitively binding miRNAs to regulate downstream mRNA targets. To identify potential miRNAs interacting with circCNOT6L, we conducted bioinformatics analysis using circBank and mirDIP, indicating the potential association of six miRNAs with circCNOT6L (Fig. 4d). Furthermore, the analysis based on the TCGA-PRAD database suggested the significant downregulation of miR-143-5p in tumor tissues and highlighted it as a potential regulatory target of circCNOT6L (Fig. 4e). RT–qPCR outcomes indicated that both silencing and overexpression of circCNOT6L could notably modify the expression of miR-143-5p in the PC3 and LNCaP cell lines (Fig. 4f). On the basis of these findings, a luciferase reporter gene plasmid was then created to confirm the binding relationship between the two genes (Fig. 4g). The miR-143-5p sequence was mutated in this process. The luciferase assay results demonstrated that circCNOT6L negatively regulated wild type miR-143-5p while exhibiting a lack of binding with the mutant miR-143-5p (Fig. 4h). Bioinformatics analyses using miRDB, miRWalk, RNA22, TargetScan and TCGA databases identified SRSF2, EML1, TPM2 and XKR4 as potential downstream mRNA targets regulated by miR-143-5p (Fig. 4i). Coexpression analyses indicated a negative correlation between SRSF2 and miR-143-5p, prompting the selection of SRSF2 for further examination (Fig. 4j). A subsequent luciferase reporter gene plasmid assay based on SRSF2 was developed to confirm the binding relationship between the two (Fig. 4k). The luciferase assay results revealed that miR-143-5p negatively regulates the wild type SRSF2 while having no binding association with the mutant SRSF2 (Fig. 4l). Meanwhile, the overexpression of circCNOT6L significantly inhibited the binding of miR-143-5p to SRSF2 (*P* < 0.05). This finding suggests that circCNOT6L may play a crucial role in biological processes by interfering with the regulatory interaction between miR-143-5p and SRSF2 (Supplementary Fig. 3a). The western blotting results demonstrated that modifying the expression levels of circCNOT6L and miR-143-5p significantly impacted SRSF2 expression (Fig. 4m), signifying a regulatory relationship between circCNOT6L, miR-143-5p and SRSF2. The application of RNA 3D-structure prediction was used to elucidate the binding interactions between circCNOT6L and miR-143-5p, as well as between miR-143-5p and SRSF2 (Fig. 4n,o). The results above indicate that circCNOT6L can competitively bind to miR-143-5p, thereby regulating its mRNA expression. As miR-143-5p negatively regulates the expression of SRSF2, circCNOT6L positively regulates the expression of SRSF2 through miR-143-5p. To further confirm the role of circCNOT6L in regulating the expression of SRSF2 through the competing-endogenous-RNA mechanism, AGO2 RNA immunoprecipitation was performed followed by RT–qPCR analysis. The results showed that both miR-143-5p and circCNOT6L were significantly enriched in the AGO2 pull-down samples compared with the immunoglobulin G (IgG) group (Supplementary Fig. 3b–e), indicating that they are bound to the AGO2 complex. This confirms the interaction between circCNOT6L and miR-143-5p, supporting the competing-endogenous-RNA regulatory mechanism. The IHC results indicated a higher SRSF2 expression in PCa tissues than in normal tissues (Fig. 4p). Moreover, SRSF2 is highly expressed in PCa tissues with a high risk in contrast to those with a low risk (Fig. 4q). Overall, the findings imply a close association between SRSF2 and the malignant progression of PCa. Furthermore, the expression level of circCNOT6L in patient tissues positively correlated with SRSF2 (Fig. 4r).

**Fig. 5:**
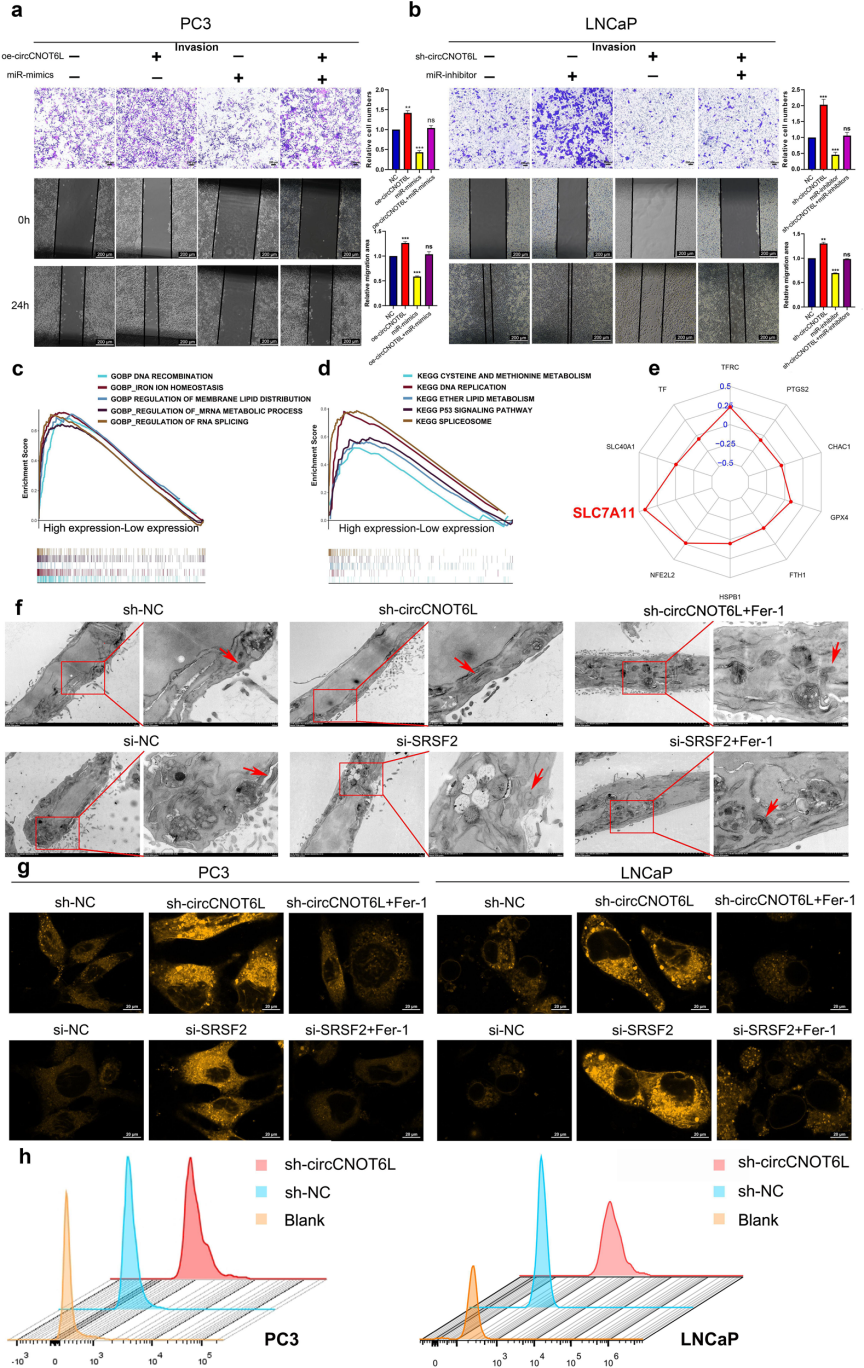
circCNOT6L positively regulates SCL7A11 by affecting SRSF2 and inhibits ferroptosis of PCa cells. **a**, **b** Transwell invasion and wound-healing assays demonstrated that circCNOT6L coregulated PCa cell invasion and migration through the miR-143-5p–SRSF2 pathway. **c**, **d** GSEA revealed potential functions of SRSF2 and signalling pathways involved. **e** Coexpression analysis results demonstrate the relevance of SRSF2 to pivotal regulatory mRNAs for ferroptosis. **f** Transmission electron microscopy assay was used to compare the mitochondria of PC3 cells treated with sh-NC, sh-CNOT6L and Fer-1, and observe the morphological differences of mitochondria in different groups. **g** The distribution of Fe^2+^ in PC3 and LNCaP cells treated with a fluorescent probe was observed using confocal microscopy. **h** C11 probe was used to assess lipid ROS by flow cytometry. ^*^P < 0.05, ^**^P < 0.01, ^***^P < 0.001.

Wound-healing and transwell rescue assays revealed that overexpressing circCNOT6L alleviated the inhibitory effects of miR-143-5p on cell migration and invasion (Fig. 5a,b). Furthermore, miR-143-5p exhibited a strong and negative association with PFI among patients with PCa (Supplementary Fig. 2a), correlating negatively with the T stage, Gleason score and N stage. Notably, miR-143-5p was highly expressed in patients who underwent postoperative androgen deprivation therapy (Supplementary Fig. 2b). Gene set variation analysis highlighted the potential biological functions and molecular pathways influenced by miR-143-5p (Supplementary Fig. 2c,d). Survival analysis demonstrated a significant positive correlation between SRSF2 expression and PFI in patients with PCa (Supplementary Fig. 2e). Differential expression analysis indicated elevated SRSF2 levels in tumor tissues compared with normal tissues (Supplementary Fig. 2f) and in metastatic castration-resistant PCa compared with nonmetastatic castration-resistant PCa tissues (Supplementary Fig. 2g). Clinical correlations revealed a significant positive relationship between SRSF2 and T stage, Gleason score and N stage, with nonresponsive patients displaying higher SRSF2 expression (Supplementary Fig. 2h).

### EIF4A3 mediates the generation of circCNOT6L

The bioinformatics analysis conducted in this study successfully predicted the presence of the RNA-binding protein EIF4A3, which has been found to bind to the reverse complementary sequence known as the Alu sequence both upstream and downstream of circCNOT6L. This prediction was further validated through an RNA immunoprecipitation experiment, which demonstrated the binding of EIF4A3 to the Alu sequences associated with CNOT6L precursor (Supplementary Fig. 3f,g). Subsequently, the relationship between EIF4A3 and circCNOT6L was investigated, and RT–qPCR analysis revealed that the knockdown of EIF4A3 resulted in a downregulation of circCNOT6L expression (Supplementary Fig. 3h). Furthermore, the results obtained from the RT–qPCR assay indicated that the knockdown of EIF4A3 led to a decrease in SRSF2 expression while simultaneously increasing the expression of mir-143-5p (Supplementary Fig. 3i–k). The aforementioned result was further confirmed using TCGA-PRAD datasets (Supplementary Fig. 3l).

### circCNOT6L positively regulates SCL7A11 by affecting SRSF2 and inhibits ferroptosis of PCa cells

**Fig. 6:**
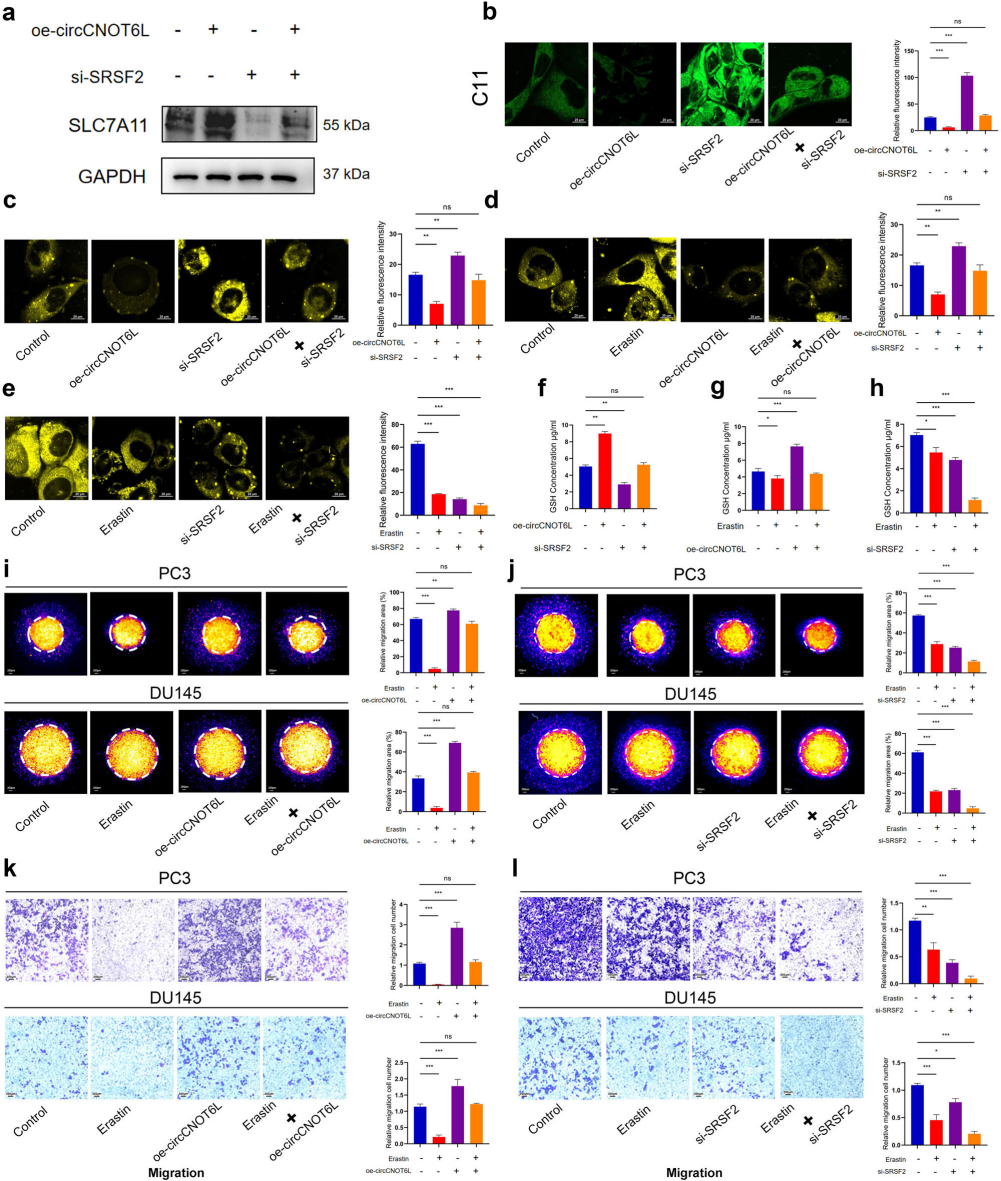
circCNOT6L inhibits cellular ferroptosis via the SRSF2–SLC7A11 axis and promotes malignant progression in PCa. **a** Western blotting reveals regulatory relationships between circCNOT6L, SRSF2 and SLC7A11. **b** Lipid ROS changes assessed by confocal using the C11 probe after treatment of cells with oe-circCNOT6L and si-SRSF2. **c**–**e** Following treatment with oe-circCNOT6L, si-SRSF2 and erastin (5 μM), the distribution of Fe²⁺ in PC3 and LNCaP cells was visualized using confocal microscopy with fluorescent probes. **f**–**h** PC3 and DU145 were treated with oe-circCNOT6L, si-SRSF2 and erastin (5 μM), and cellular GSH content was measured using a GSH assay kit and zymography. **i**, **j** Changes in cell-invasion ability were assessed using a 3D Matrigel drop invasion assay following treatment of PC3 and DU145 cells with oe-circCNOT6L, si-SRSF2 and erastin (5 μM). **k**, **l** Changes in cell-migration ability were assessed using a transwell assay following treatment of PC3 and DU145 cells with oe-circCNOT6L, si-SRSF2 and erastin (5 μM).

We investigated the role of SRSF2 in PCa progression through GSEA, revealing its association with iron and lipid metabolism (Fig. 5c,d), suggesting a potential role in ferroptosis regulation in PCa. Correlation analysis of seven ferroptosis-regulated genes with SRSF2 demonstrated a significant positive correlation between SRSF2 and SLC7A11 (Fig. 5e). Biological projection electron microscopy uncovered specific changes in ferroptosis PC3 mitochondria following circCNOT6L and SRSF2 knockdown, including mitochondrial swelling, cristae deletion and increased mitochondrial membrane density. These changes were reversed by a ferroptosis inhibitor (Fer-1) (Fig. 5f). The intracellular ferrous ion content was observed by confocal microscopy using the ferrous ion probe, revealing a significant increase in ferrous ions in PCa cells (PC3 and LNCaP) after knockdown of circCNOT6L and SRSF2 (Fig. 5g). This increase in ferrous ions indicated the activation of the ferroptosis mechanism, which could be rescued by Fer-1. Furthermore, circCNOT6L knockdown increased intracellular lipid ROS, as assessed using flow cytometry (Fig. 5h). Furthermore, western blotting and flow cytometry analysis revealed that neither circCNOT6L knockdown nor overexpression had any impact on apoptosis or autophagy (Supplementary Fig. 4a–c). In addition, no correlation was observed between SRSF2 and p62 expression in the TCGA-PRAD cohort (Supplementary Fig. 4d). These results suggested that circCNOT6L inhibits ferroptosis of PCa cells by regulating SRSF2, which in turn promotes malignant PCa progression. To elucidate the regulatory relationship between circCNOT6L, SRSF2 and SLC7A11, western blotting was conducted. The results demonstrated that circCNOT6L positively regulates SLC7A11 protein expression via SRSF2 (Fig. 6a). To further confirm the role of the circCNOT6L–SRSF2 axis in ferroptosis, C11 and Fe²⁺probes were used, showing that this axis negatively regulates cellular ferroptosis (Fig. 6b,c). Rescue experiments with erastin, a known SLC7A11 inhibitor, further validated that circCNOT6L inhibits ferroptosis in PCa cells by upregulating SLC7A11 (Fig. 6d,e). GSH assays further confirmed that circCNOT6L suppresses ferroptosis in PCa cells through the SRSF2–SLC7A11 axis (Fig. 6f–h). Moreover, 3D Matrigel drop invasion and transwell assays provided compelling evidence that the circCNOT6L–SRSF2–SLC7A11 axis promotes PCa cell invasion and migration by regulating ferroptosis, ultimately contributing to the malignant progression of PCa (Fig. 6i–l).

### SRSF2 inhibits ferroptosis by regulating differential splicing of SLC7A11

**Fig. 7:**
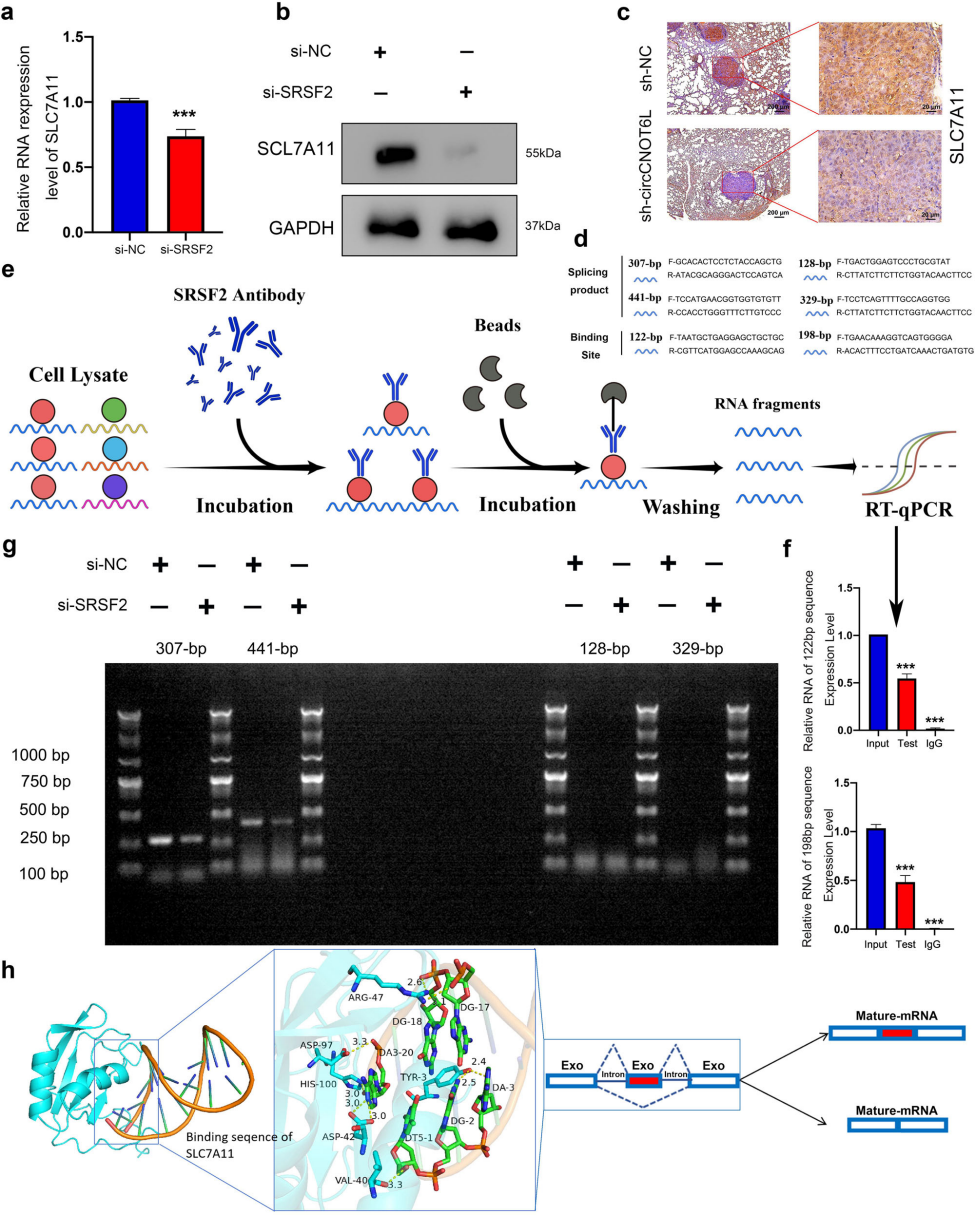
SRSF2 inhibits ferroptosis by regulating differential splicing of SLC7A11. **a** RT–qPCR was used to verify the correlation between SRSF2 and SLC7A11 at the transcriptional level. **b** Western blotting was used to verify the correlation between SRSF2 mRNA and SLC7A11 at the protein-expression level. **c** The correlation between circCNOT6L and SLC7A11 at the expression level was verified by IHC in tissues from nude-mouse lung metastases. **d** Online bioinformatics tools (ESEfinder) were used to design two potential binding sequences and four possible mature-body sequences. **e** Schematic illustration of RNA pull-down for SRSF2. **f** RT–qPCR was used to validate the results of the RNA immunoprecipitation. **g** Nucleic acid electrophoresis experiments was used to validate the expression of mRNA from a sheared fragment of SLC7A11. **h** 3D structure predicts the binding site of SRSF2 protein to SLC7A11 mRNA and resolves the form in which SRSF2 regulates variable shearing of SLC7A11. ^*^P < 0.05, ^**^P < 0.01, ^***^P < 0.001.

Furthermore, we used RT–qPCR with western blotting to investigate the regulatory relationship between SRSF2 and SLC7A11, and the results indicated that knockdown of SRSF2 reduced the transcriptional level and protein expression of SLC7A11. Furthermore, western blotting revealed that protein levels of SLC7A11 are also reduced after knockdown of circCNOT6L (Fig. 7a,b and Supplementary Fig. 4e). IHC results from mouse lung metastases suggested a positive correlation between circCNOT6L and SRSF2 expression (Fig. 7c). This further indicated that SRSF2 may act as a shearer, regulating the differential splicing of SLC7A11 and promoting the production of mature-body SLC7A11. To further clarify the site where SRSF2 shears SLC7A11, we designed two potential binding sequences and four possible mature-body sequences using an online bioinformatics tool (ESEfinder) (Fig. 7d).

Moreover, RNA immunoprecipitation experiments confirmed the binding relationship between the SRSF2 protein and the binding sequences (Fig. 7e). The RT–qPCR results showed two binding splice sites for SRSF2 (122-bp and 198-bp) on the exon of SLC7A11 (Fig. 7f). Nucleic acid electrophoresis experiments demonstrated that SRSF2 knockdown reduced the production of mature SLC7A11 mRNA (441-bp and 307-bp) (Fig. 7g). The application of RNA 3D-structure prediction was used to elucidate the binding interactions between SRSF2 and SLC7A11-precursor mRNA. Our results revealed that SRSF2 binds directly to the region between the two 5′ splice sites of SLC7A11, promoting the production of mature SLC7A11 bodies (Fig. 7h). Survival analysis based on the TCGA-PRAD data indicated that patients with PCa with high SLC7A11 and SRSF2 expression had significantly poorer prognostic outcomes than other patients (Supplementary Fig. 5a). A nomogram, including Gleason score, pathological T stage, pathological N stage, SRSF2 and SLC7A11, accurately predicted the 1, 3 and 5 year PFI of patients with PCa (Supplementary Fig. 5b,c).

**Fig. 8:**
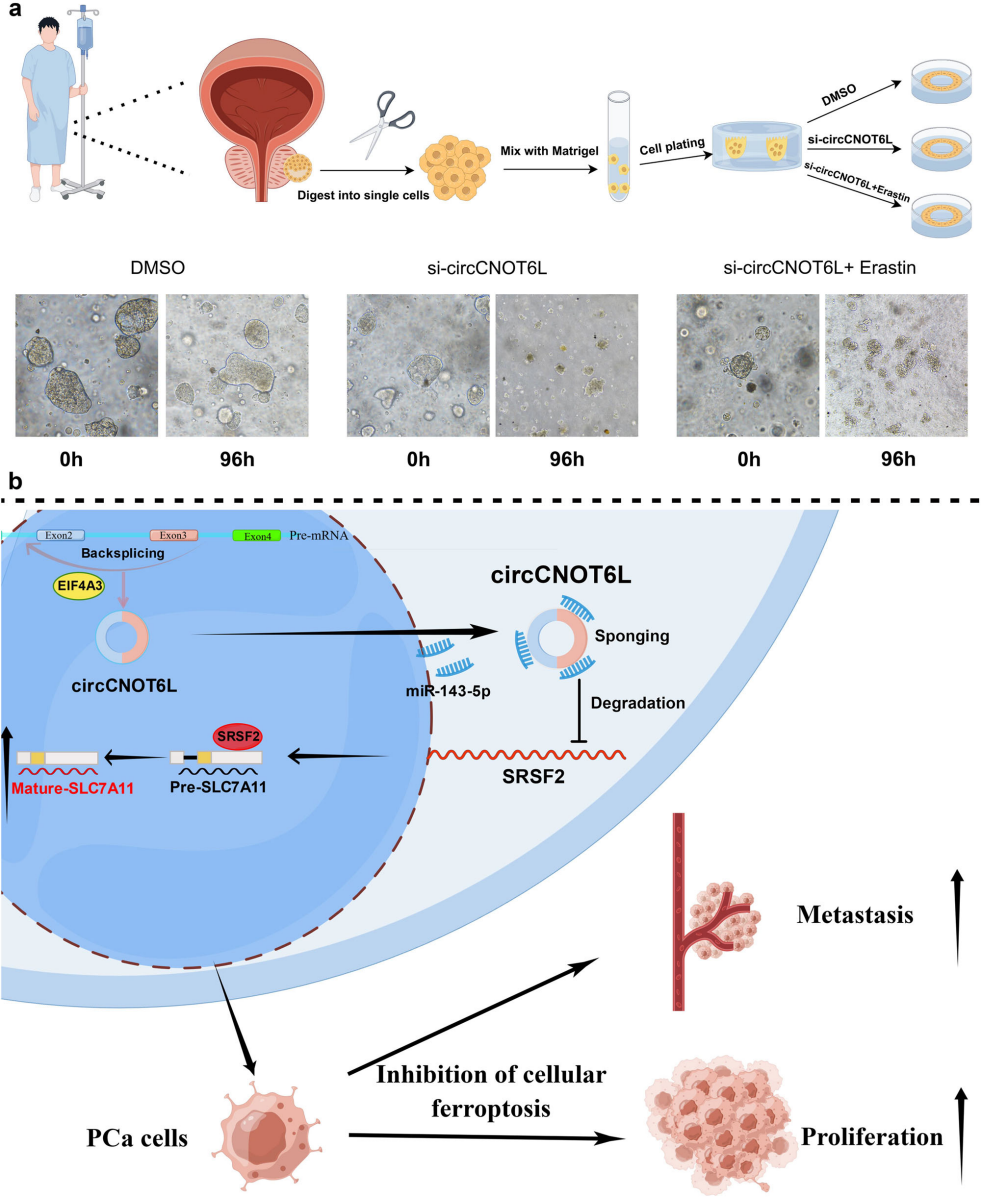
Determining the potential of circCNOT6L as a therapeutic target at the organoid level. **a** The possibility of using circCNOT6L-si in combination with erastin to treat PCa was evaluated by analyzing the growth of circCNOT6L-si in comparison to organoids in the si-circCNOT6L+erastin group after dimethyl sulfoxide treatment. **b** The proposed model of the mechanism of circCNOT6L promoting PCa malignant progression.

Finally, PCa organoids were used to study the effects of si-circCNOT6L with ferroptosis activator on tumor cells. The results demonstrated significant inhibition of PCa organoid growth with si-circCNOT6L alone compared with the control group. This effect of disintegration of PCa organoids was further enhanced after treatment with si-circCNOT6L and the ferroptosis activator erastin. These results suggest that reducing the expression level of circCNOT6L, along with the ferroptosis activator, can significantly inhibit the malignant progression of PCa (Fig. 8a). The proposed mechanism by which circCNOT6L promotes PCa malignancy is illustrated in Fig. 8b.

## Discussion

The presence of positive lymph nodes following radical prostatectomy represents a detrimental pathological feature that significantly impacts the overall well-being and life expectancy of patients with PCa^22^. Metastatic cells often evade established programmed-cell-death mechanisms, including apoptosis, autophagic cell death, necrosis and ferroptosis^23,24^, posing challenges in identifying effective therapeutic targets for distant metastases in PCa^3^. Consequently, it is imperative to investigate the pivotal regulatory factors and molecular mechanisms governing distant metastasis to develop effective treatment strategies for PCa. CircRNAs, characterized by their covalently closed structure, have emerged as stable and more resistant entities with potential applications as diagnostic and therapeutic biomarkers^6,25^. Dysregulation of circRNAs has been implicated in various cancers, influencing processes such as growth invasion, vascularization and apoptosis^26^. For instance, Huang et al. demonstrated that circ_0006168 (circCNOT6L) promotes tumorigenesis in esophageal squamous cell carcinoma^27^, and Wang et al. showed that it promotes the proliferation of human glioblastoma cells^28^. These findings provide evidence for the involvement of circCNOT6L in various types of cancer. However, the function of circCNOT6L in PCa remains unexplored. Our study provides evidence that circCNOT6L expression is significantly elevated in lymph nodes affected by mPCa compared with tumor tissues. Furthermore, a positive correlation between circCNOT6L expression and metastasis was observed in patients with PCa with a high Gleason score. In vivo experiments demonstrated that circCNOT6L downregulation effectively inhibits lung metastasis and reduces subcutaneous tumor growth in nude mice. Moreover, in vitro investigations uncovered the regulatory role of circCNOT6L in the proliferation, invasion and metastasis of PCa cells, highlighting its potential as a therapeutic target for distant metastasis of PCa. Xu et al. reported that miR-143-5p could inhibit breast cancer progression through the GLUT1 pathway^29^, whereas He et al. demonstrated that it promoted gallbladder carcinogenesis through HIF-1^30^. In addition, miR-143-5p has been shown to play a role in gastric and pancreatic carcinogenesis^31^, indicating its involvement in the pathogenesis of various cancers. Our study establishes a significant negative correlation between miR-143-5p and circCNOT6L. Our work further elucidated the mechanistic role of circCNOT6L in upregulating SRSF2 expression by competitively sequestering miR-143-5p, which is known to target SRSF2. SRSF2, recognized as a shearosome, has been previously associated with promoting tumorigenesis in hepatocellular carcinoma^32^. Liang et al. further established that SRSF2 promotes tumorigenesis by inhibiting hematopoietic differentiation^33^. Our research uncovered that RNA-binding protein EIF4A3 facilitated the biogenesis of circCNOT6L and contributed to PCa metastasis by binding to the Alu regions of a precursor mRNA of CNOT6L.

Studies have shown the significance of ROS in the process of ferroptosis^34^. Inhibition of SLC7A11, GPX4 and others can result in ROS accumulation, which, in turn, can promote cell oxidative death, ultimately leading to ferroptosis^35,36^. Evidence has demonstrated that ferroptosis caused by ROS accumulation is present in tumors^13^, and underscores the critical role of ferroptosis in cancer invasion and metastasis. Li et al. demonstrated that CST1 promotes gastric cancer metastasis by inhibiting ferroptosis, suggesting that the regulation of ferroptotic pathways can influence tumor progression^37^. Similarly, Liu et al. found that GPX4 knockout mitigates the tumorigenic and metastatic activity heightened by 27HC-resistant cells, further linking ferroptosis resistance with enhanced malignancy^38^. Moreover, Schwab et al. discovered that various forms of EMT activation increase cancer-cell sensitivity to ferroptosis, highlighting ferroptosis as a pivotal player in the EMT process^39^. Collectively, these findings emphasize the intricate interplay between ferroptosis and EMT, offering valuable insights into potential strategies for mitigating cancer metastasis by modulating ferroptotic pathways. Meanwhile, the interplay between ROS and PCa progression has been well documented, with PMANs hindering the expression of GPX4 and SLC7A11, leading to ROS accumulation and iron dysregulation in PCa^40^. Ghoochani et al. showed that treatment with erastin, an SLC7A11 inhibitor, significantly reduced PCa invasion and migration both in vitro and in vivo, reinforcing the potential of targeting ferroptosis pathways for therapeutic interventions^41^. Moreover, CEMIP supports the survival of detachment-resistant PCa cells by modulating SLC7A11 expression, decreasing ROS levels and blocking iron metabolism. GSEA enrichment analysis further indicated a potential association between circCNOT6L and SRSF2 in the ferroptosis process. Furthermore, our in vitro experiments underscored the ability of circCNOT6L and SRSF2 to modulate ferrous iron metabolism, mitochondrial morphology and ROS accumulation in PCa cells. Subsequent mechanistic investigations revealed a positive correlation between SRSF2 and SLC7A11 at both the transcriptional and protein levels. Notably, SLC7A11 plays a crucial role as an inhibitory molecule in the ferroptosis mechanism^42^. Wang et al. found that SLC7A11 played an important role in pancreatic ductal adenocarcinoma and that pharmacological inhibitors of SLC7A11 were effective in inducing tumor regression and inhibiting metastasis in this disease^43^. Sun and colleagues discovered that SLC7A11 has the ability to influence ferroptosis mechanisms that impact chemoresistance in PCa^19^. Our mechanistic studies unveiled the regulatory role of SRSF2 in controlling the variable splicing of SLC7A11, thereby enhancing SLC7A11 expression and preventing ferroptosis in PCa cells. Finally, we determined that the combination of the ferroptosis activator and circCNOT6L knockdown was effective in halting the growth of PCa organoids. In conclusion, our study elucidates novel insights by which circCNOT6L acts as an miR-143-5p sponge to activate SRSF2, thereby regulating the expression of SLC7A11 and impeding ferroptosis in PCa, ultimately promoting its malignant progression. Our research provides novel insights into the crucial role of EIF4A3-mediated circCNOT6L in PCa cells and the unique involvement of the circCNOT6L–SRSF2–SLC7A11 axis in the distant metastasis of PCa. These findings suggest that circCNOT6L may serve as a promising therapeutic target for patients with PCa. In addition, the exploration of combinatorial approaches, such as circCNOT6L-siRNA with ferroptosis activator, holds promise for advancing PCa treatment strategies.

## Supplementary information

Supplementary information

Raw Data

## Data Availability

The data and materials in the current study are available from the corresponding author on reasonable request.
