# Supplementary material for: CircCNOT6L modulates alternative splicing of SLC7A11 via splicing factor SRSF2 to confer ferroptosis resistance and promote metastasis in prostate cancer
Source: Exp Mol Med. 2025 Sep 29;57(9):2106–20. doi: 10.1038/s12276-025-01540-y (PMC12508045; doi:10.1038/s12276-025-01540-y)
Supplement: Supplementary file 1 — Supplementary information [file 12276_2025_1540_MOESM1_ESM.pdf]

Supplementary documents

Supplementary materials and methods

Quantitative real-time PCR

Total RNA RT-qPCR was extracted using TRIzol reagent (Invitrogen, USA). Reverse Transcription into cDNA was done using HiScript III 1st strand cDNA synthesis kit (Vazyme, China). RT-qPCR was performed using ChamQ SYBR qPCR Master Mix Kit (Vazyme, China). GAPDH was used as a control for normalizing the relative mRNA levels. Results were analyzed using the 2-Ct method. Primer sequences are shown in Supplementary table 3.

Cell Proliferation assay

Cell proliferation was checked with Cell counting kit-8 (CCK8) (Yeason Biotech Co., Ltd, Shanghai, China). Colony formation assays were also performed to check cell proliferation.

Wound healing assay

The cells were cultured in 6-well plates. When the confluency reached 100 percent, scratches were made, and images were taken at 0 and 24 hours. Calculation of the 24 h migration distance was done by subtracting the distance between the scratch edge at 0 h and the migration edge at 24 h using ImageJ software. All the experiments were performed in triplicate.

Migration and invasion assay

This experiment was performed in transwell plate with multipolar 8.0 μl

polycarbonate membrane (Corning, USA). Cells with mixed with serum free medium and kept in upper chamber. And 500 μl of complete medium was added to lower chamber. Then cells were incubated at 37°C with 5% CO2. After 48 hours cells with stained with crystal violet and photos taken using 100X magnification. Each experiment was performed in triplicate.

luciferase reporter assay

The circRNA and miRNA association was predicted using circbank, circAtlas and interactome. The sequence of wild and mutant types of circCNOT6L were synthesized by company(Aibosi, Shanghai, China). Luciferase activity was measured using a dual-luciferase reporter gene kit (Yeasen, Shanghai, China). All the experiments were performed in triplicate.

Western blot

Total protein was extracted using SDS lysis buffer (Beyotime, Nantong, China). Total protein as calibrated using BCA protein Assasy kit (Beyotime, Nantong, China). Protein (40μl per lane) was loaded into 12% SDS gel. Polyvinylidene fluoride (PVDF) membranes (Millipore, Burlington, MA, USA) were used for transfer. 5% milk was used as non specific block. And then primary antibodies were used and incubated overnight at 4 °C. The next day, it was washed three times 10 mins each with PBST. Then secondary antibodies were used and incubated for 1 hour at room temperature. It was again washed with PBST for 10 mins each for thrice. Detecting the protein bands was done using a silver stain detection system (Beyotime, Nantong, China). The detail of antibody used in this study was shown in Supplementary table 4.

Flurorescnece in situ hybridization (FISH)

The sublocation of circCNOT6L in the cell was detected via FISH kit (BIS-P0001,Guangzhou Boxin Biotechnology Co., Ltd., Guangzhou, China) following the manufacturer's instruction. This experiment was performed three times using a Zeiss LSM880NLO confocal microscope (2 + 1 with BIG, Leica Microsystems, Wiezlar, Germany).

RNA immunoprecipitation (RIP)

The RIP assay was conducted using a RIP kit (Guangzhou Boxin Biotechnology Co., Ltd.). Following the manufacturer's guidelines, $1x10^7$ PCa cells were subjected to immunoprecipitation using RIP buffer containing SRSF2-coupled magnetic beads. The amplification of RNA from the RNA-protein complexes was verified through qPCR, with IgG serving as the negative control.

Immunohistochemistry (IHC) analysis

4% ParaformAldehyde (PFA) was used for the fixation and embedding of tissues. The tissue sections were then incubated with antibodies overnight at 4°C after deparaffinization, dehydration, antigen retrieval, and blocking. After incubating with biotinylated goat anti-rabbit IgG for 20 minutes at RT, the tissue sections were incubated with streptavidin-horseradish peroxidase for 30 mins. As a final step, the tissues were stained with diaminobenzidine-H2O2 and hematoxylin.

Enrichment Analysis

Biological pathways between clusters were explored by gene set variation analysis (GSVA). GO (Gene Ontology) describes biological processes(BP), molecular

functions(MF), and cellular components(CC) of genes The Kyoto Encyclopedia of Genes and Genomes (KEGG) were used to annotate involved pathways. Furthermore, the FDR threshold (0.05) was applied to c5.cp.kegg.v5.2.symbols.gmt, which is the reference gene set.

Identification of mRNAs with differential expression

EDGER R package (http://bioconductor.org/package/EDGER/) was applied to screen the differential expressed genes (DEGs) between normal and tumor samples in the GSE179312 and tissue Chip. After normalization of the raw data, the differences of genes expression between two types of samples were analyzed with false discovery rate (FDR) < 0.01 and log2 |fold change| > 1.

Nomogram development and validation

Univariate and multivariate Cox regression analyses were used to construct nomograms to predict the impact of target genes and other clinicopathological features on prognosis and metastasis in PCa patients. The Nomogram was validated by the calibration plots and consistency index (C-index) using the rms package v5.1 in R (https://cran.r-project.org/web/packages/rms /index.html).

Supplementary Figures

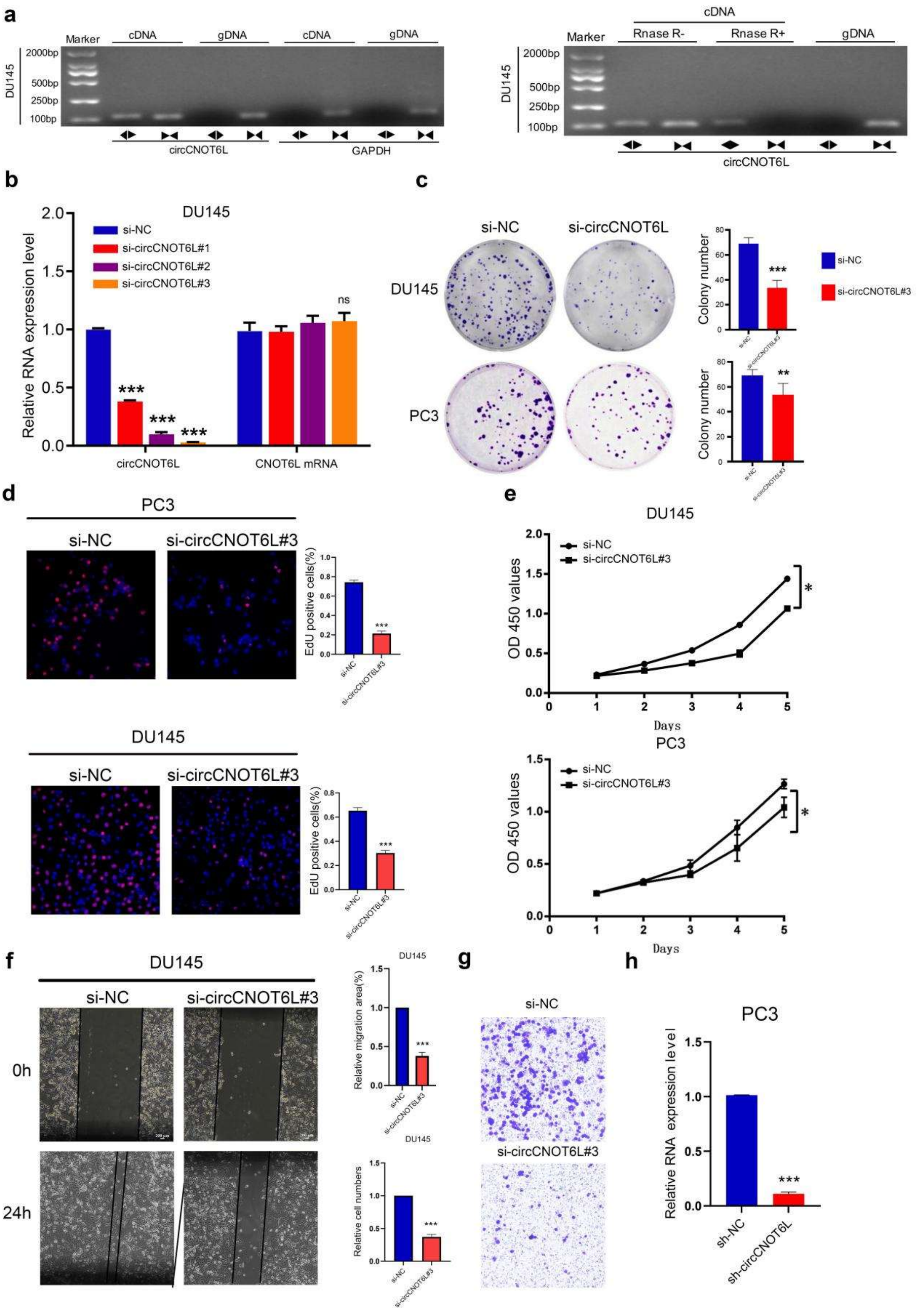

Supplementary Fig. 1. a. Northern blotting assay experiments suggested that circCNOT6L was detected in cDNA but not in the gDNA group in DU145 cell lines. **b**.The efficacy of

circCNOT6L knockdown in the DU145 cell line was measured by qPCR. **c-e**. The effect of circCNOT6L knockdown on the proliferative potential of PCa cell lines (PC3 and DU145) was evaluated by colony forming, Edu and CCK8 assays. **f-g**. The effect of circCNOT6L knockdown on the migration potential of DU145 cell lines was evaluated by wound healing and Transwell migration assay. **h**. The efficacy of sh-circCNOT6L in the PC3 cell line was measured by qPCR *$p<0.05$, **$p<0.01$, ***p

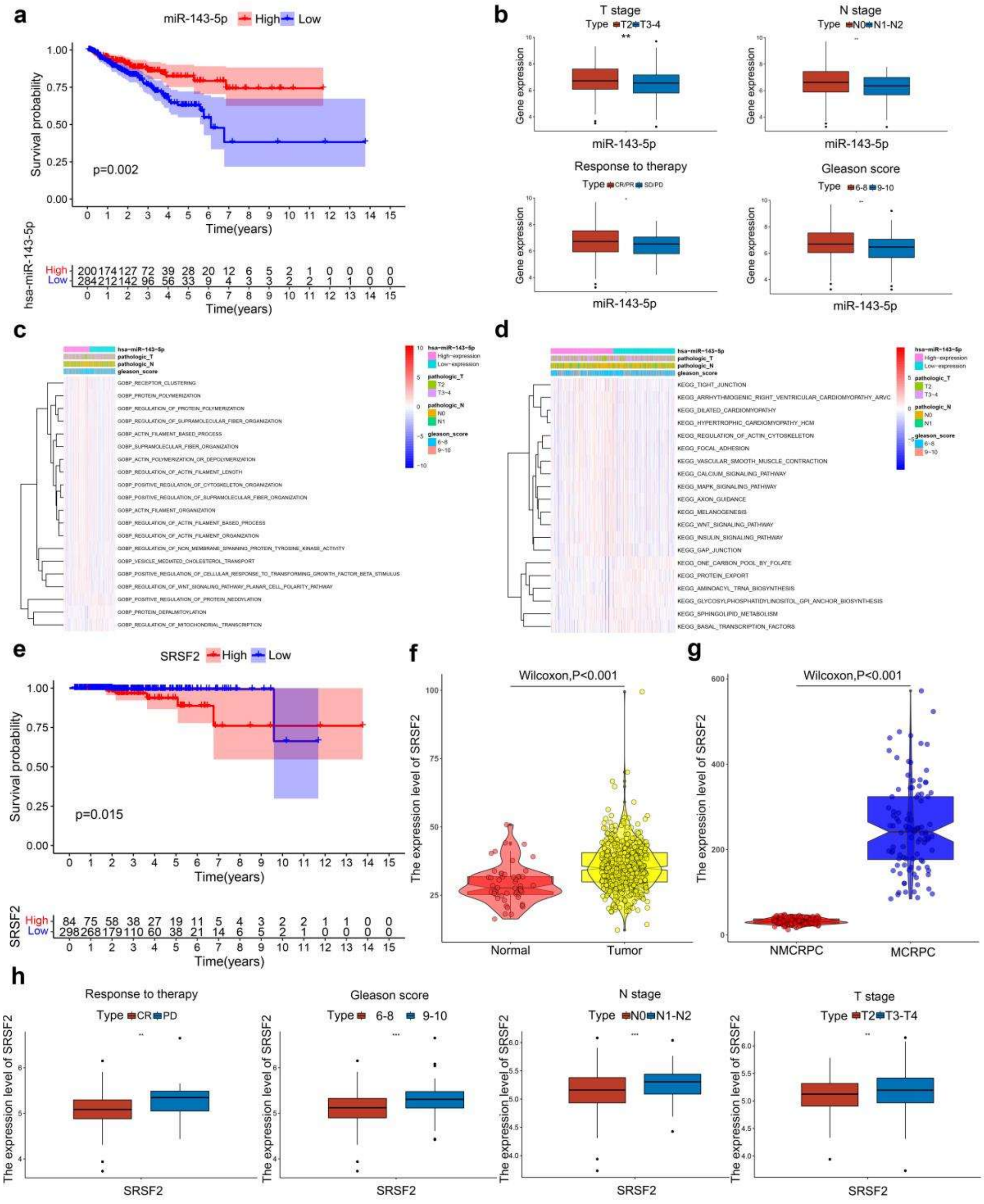

Supplementary Fig. 2. a. Survival analysis showed the effect of miR-143-5p on PFI in PCa

patients. **b**. Correlation analysis showed the correlation between miR-143-5p and clinical characteristics of PCa patients. **c-d**. GSVA results showed the possible cellular functions and pathways involved by miR-143-5p. **e**. Survival analysis showed the effect of SRSF2 on PFI in PCa patients. **f-g**. Violin plots showing the expression of SRSF2 between normal and tumour tissues and between NMCRPC and MCRPC. **h**. Correlation analysis showed the correlation between SRSF2 and clinical characteristics of PCa patients.*p<0.05, **p<0.01, ***p<0. 001.

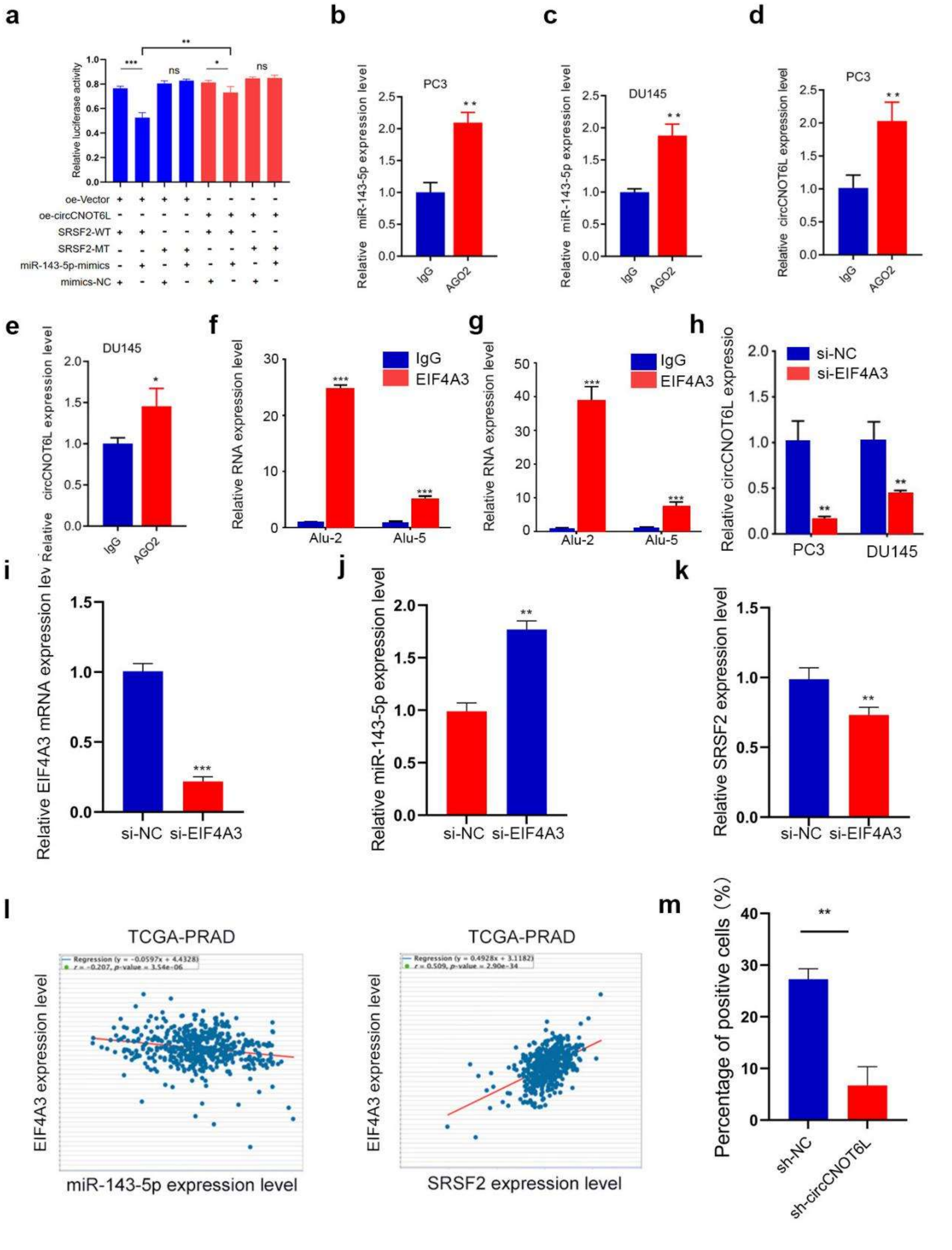

Supplementary Fig. 3. a. Dual-luciferase reporter assay designed to investigate whether circCNOT6L overexpression affects the binding of miR-143-5p to SRSF2. **b-e.** The interaction between miR-143-5p and circCNOT6L was analyzed using RIP with an AGO2 antibody, followed by qPCR to detect their expression levels. **f-g.** RNA-IP assay to verify that EIF4A3 binds to the alu sequence of circCNOT6L. **h.** qPCR results showing changes in circCNOT6L expression after knockdown of EIF4A3. **i-k.** The efficiency of knocking down EIF4A3 was detected by qPCR, as well as changes in the expression levels of circCNOT6L as well as miR-143-5p and SRSF2. **l.** Correlation analysis based on the TCGA-PRAD cohort shows correlation between EIF4A3 with miR-143-5p and SRSF2. **m.** Statistical analysis of PCNA-positive cells in IHC staining.*p<0.05, **p<0.01, ***p<0. 001.

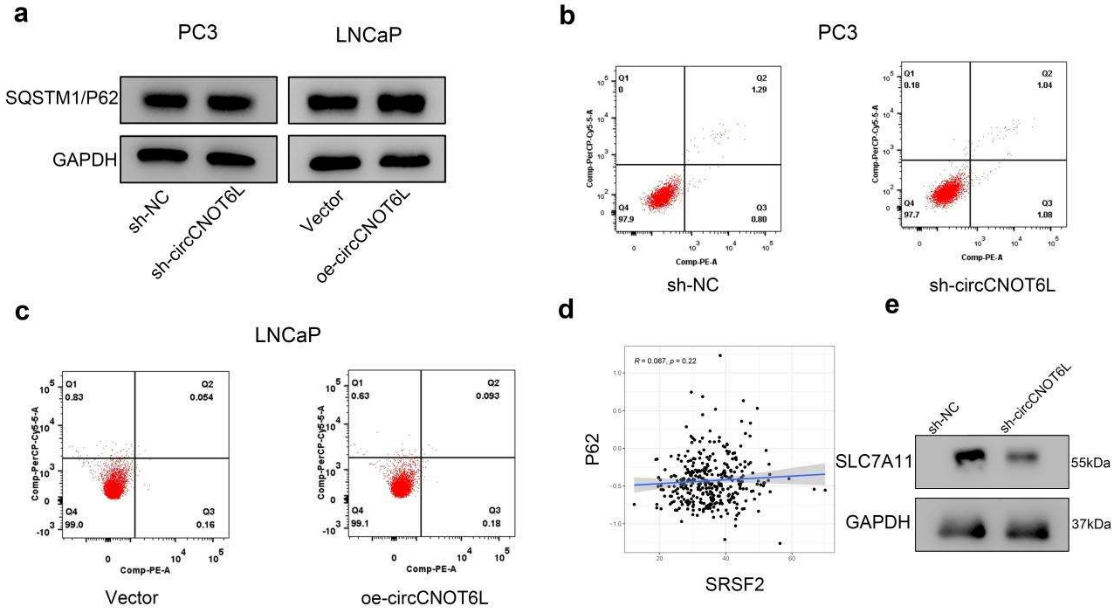

Supplementary Fig. 4. **a**. Western blot revealed alterations in the expression of autophagy-related proteins following transcriptional modulation of circCNOT6L. **b-c**. The result of Flow cytometry shows alteration in apoptosis after altering circCNOT6L expression at the transcriptional leve in PC3 and LNCaP cell lines. **d**. Correlation analysis` based on the TCGA-PRAD cohort showed correlation between SRSF2 and autophagy-related proteins. **e**. Western blotting describes changes in protein levels of SLC7A11 after knockdown of circCNOT6L.*p<0.05, **p<0.01, ***p

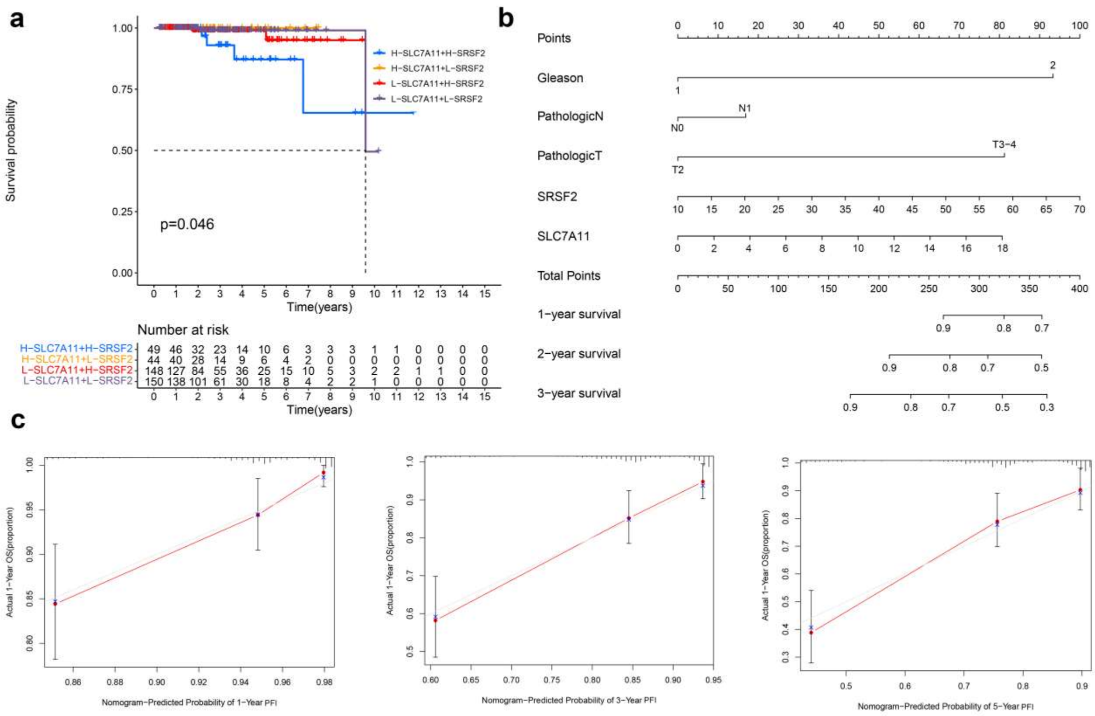

Supplementary Fig. 5. **a**. KM survival analysis of patients with PCa according to SRSF2+SLC7A11 score. **b**. Nomogram to predict theprobability of 1-, 3-, and 5-year PFI. **c**.Calibration plots of the Nomogram were used to predictthe probability of 1-, 3-, and 5-year PFI.

Supplementary Tables

Patients	PSA	Gleason Score	TNM	Age	Date of surgery
B39933	10.99	4+5=9	T2cN1M0	61	20180731
B35805	15.37	4+5=9	T3aN1M0	78	20180717
B33972	184.4	5+4=9	T4N1M0	64	20180710
A87314	32.2	4+5=9	T4N1M1b	70	20180123
B31301	7.64	3+4=7	T3aN1M0	74	20180717

Supplementary Table 1:The clinical information of patients.

circRNA_id	up_down	log^2FoldChange	P-Value
hsa_circ_0001971	Up	4.13274998	2.62E-09
hsa_circ_0006168	Up	3.036023506	3.30E-08
hsa_circ_0004650	Up	4.671019414	2.74E-06
hsa_circ_0001860	Up	2.793550278	1.22E-05
hsa_circ_0000110	Up	2.671213655	1.70E-05
hsa_circ_0001708	Up	3.088023424	7.33E-05
hsa_circ_0000386	Up	2.558213655	7.79E-05
hsa_circ_0001069	Up	3.773700912	0.000140754
hsa_circ_0007342	Up	3.228060921	0.000302802
hsa_circ_0000479	Up	2.229281032	0.000334303
hsa_circ_0069399	Up	2.594844993	0.000386498
hsa_circ_0000992	Up	1.87110343	0.002807112
hsa_circ_0000398	Up	1.832753293	0.003180899
hsa_circ_0007195	Up	3.037218082	0.003871253
hsa_circ_0087391	Up	2.477004654	0.005429625
hsa_circ_0007854	Up	2.040578419	0.005740237
hsa_circ_0002722	Up	2.117444402	0.005943452
hsa_circ_0001451	Up	1.044194226	0.00613189
hsa_circ_0001432	Up	1.935731105	0.007663318
hsa_circ_0002077	Up	2.595196981	0.008058401
hsa_circ_0008712	Up	2.444804886	0.008257099
hsa_circ_0003380	Up	2.385766524	0.008423752
hsa_circ_0017092	Up	2.505282744	0.00871046
hsa_circ_0000268	Up	2.064160017	0.010243924
hsa_circ_0000128	Up	1.661125507	0.011649785

hsa_circ_0001314	Up	1.23152393	0.01347971
hsa_circ_0001555	Up	2.823954125	0.01598161
hsa_circ_0001789	Up	1.49163921	0.019134449
hsa_circ_0004372	Up	1.695663476	0.020728478
hsa_circ_0074944	Up	2.532346488	0.0215041
hsa_circ_0001439	Up	1.035793191	0.024184688
hsa_circ_0000195	Up	1.32786077	0.024958332
hsa_circ_0081873	Up	2.067944321	0.04333444
hsa_circ_0005432	Up	2.408319671	0.045674842
hsa_circ_0016866	Up	2.373869838	0.046600341
hsa_circ_0005142	Up	1.380599709	0.047804379

Supplementary Table 2. Differential expression of intersecting genes in our centre's data

Name	Sequence
circCNOT6L	F:5’-GGTTTGAAAGACTAATAGGGATGCC-3’<br>R:5’-TGTCATTTAGGTGCAGCGCT-3’
GAPDH	F:5’-GGGAAATTCAACGGCACAGT-3’<br>R:5’-AGATGGTGATGGGCTTCCC-3’
SLC7A11	F: 5’- TCTCCAAAGGAGGTTACCTGC-3’<br>R:5’- AGACTCCCCTCAGTAAAGTGAC-3’
SRSF2	F:5’-AATCCAGGTCGCGATCGAAG -3’<br>R:5’-CCGAGCAGCACTCCTAATGA-3’

Supplementary Table 3: The list of Primer

Name	Brand	Number	Working concentration
GAPDH	Abcam	ab8245	1/2000
PCNA	CST	#13110	1/1000
Snail	Abcam	ab216347	1/1000
N-Cadherin	Abcam	ab76011	1/5000
Vimentin	Abcam	ab92547	1/1000 55
E-Cadherin	Abcam	ab40772	1/1000 130

SRSF2	Abcam	ab204916	1/1000 35
SLC7A11	Abcam	ab307601	1/1000

Supplementary Table 4:The detail of antibody in this experiment

Name	Sequence
si-circCNOT6L#1	Sense:5'-GUUUGAAAGACUAAUAGGGAUTT-3'<br>Anti-sense:5'-AUCCCUAUUAGUCUUUCAAACTT-3'
si-circCNOT6L#2	Sense:5'-ACUCUAGGUUUGAAAGACUAATT-3'<br>Anti-sense:5'-UUAGUCUUUCAAACCUAGAGUTT-3'
si-circCNOT6L#3	Sense:5'-GAAAGACUAAUAGGGAUGCCATT-3'<br>Anti-sense:5'-UGGCAUCCCUAUUAGUCUUUCTT-3'
sh-circCNOT6L	sense:5'-GAAAGACUAAUAGGG-3'<br>Anti-sense :5-UGGCAUCCCUAUUAGUCUUUCTT-3'
oe-circCNOT6L	5'-ACTAATAGGGATGCCAAAGGAAAAATATGATCCTCCAGA<br>TCCTCGCAGAATTTATACCATCATGTCAGCAGAGGAGGTAG<br>CCAATGGGAAAAAATCTCACTGGGCAGAATTAGAAATCTCG<br>GGTAGAGTGCGGAGCCTAAGTACATCACTTTGGTCATTGAC<br>ACACTTGACAGCGCTGCACCTAAATGACAATTACCTTAGTC<br>GCATTCCACCTGATATTGCCAAGCTTCATAATCTGGTTTACC<br>TGGATCTGTCATCCAATAAACTCAGAAGTTTACCAGCAGAA<br>CTAGGAAACATGGTGTCTCTCAGGGAATTGCTTTTAAATAA<br>CAATCTGTTACGGGTTTTGCCTTATGAACTTGGTCGGCTCTT<br>CCAGCTACAAACTCTAGGTTTGAAAG-3'
si-SRSF2	Sense:5'-CUACAGCCGCUCGAAGUCU-3'<br>Anti-sense:5'-AGACUUCGAGCGGCUGUAG-3'

Supplementary Table 5:The sequense of siRNA , shRNA and oe-RNA
