# Supplementary figures and images for: CircCNOT6L modulates alternative splicing of SLC7A11 via splicing factor SRSF2 to confer ferroptosis resistance and promote metastasis in prostate cancer
Source: Exp Mol Med. 2025 Sep 29;57(9):2106–20. doi: 10.1038/s12276-025-01540-y (PMC12508045; doi:10.1038/s12276-025-01540-y)

## Slide 1
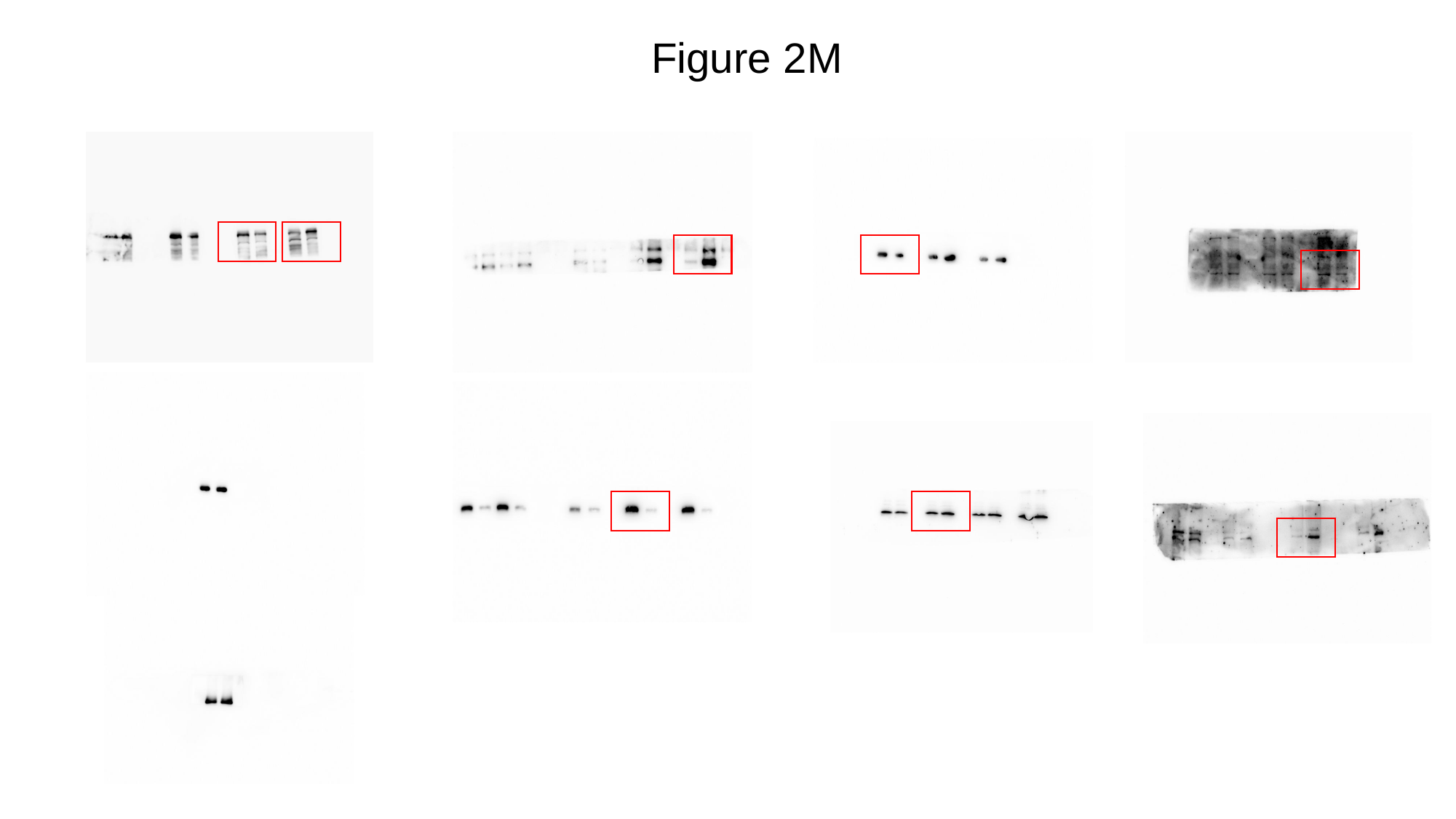

Figure 2M

## Slide 2
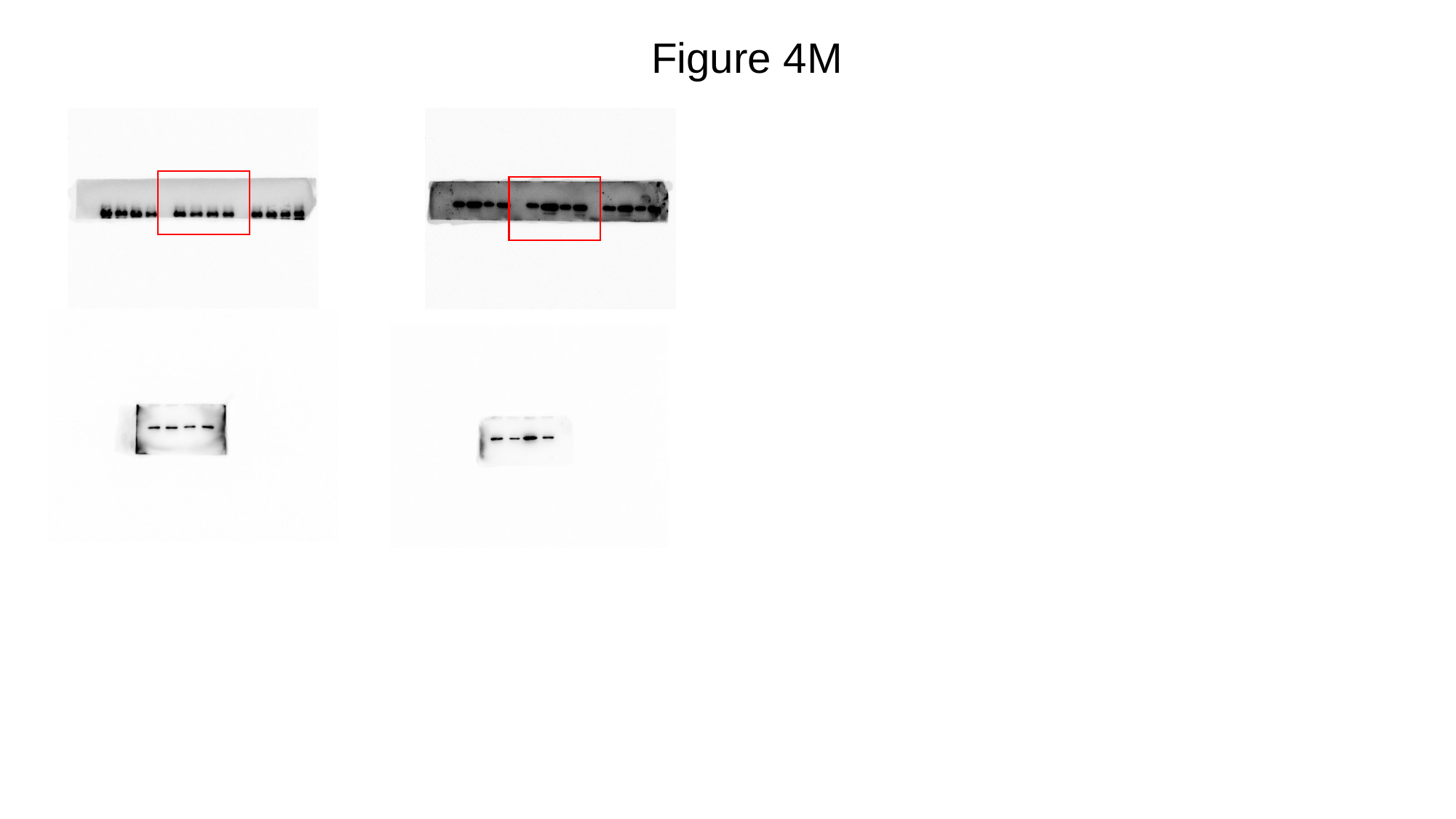

Figure 4M

## Slide 3
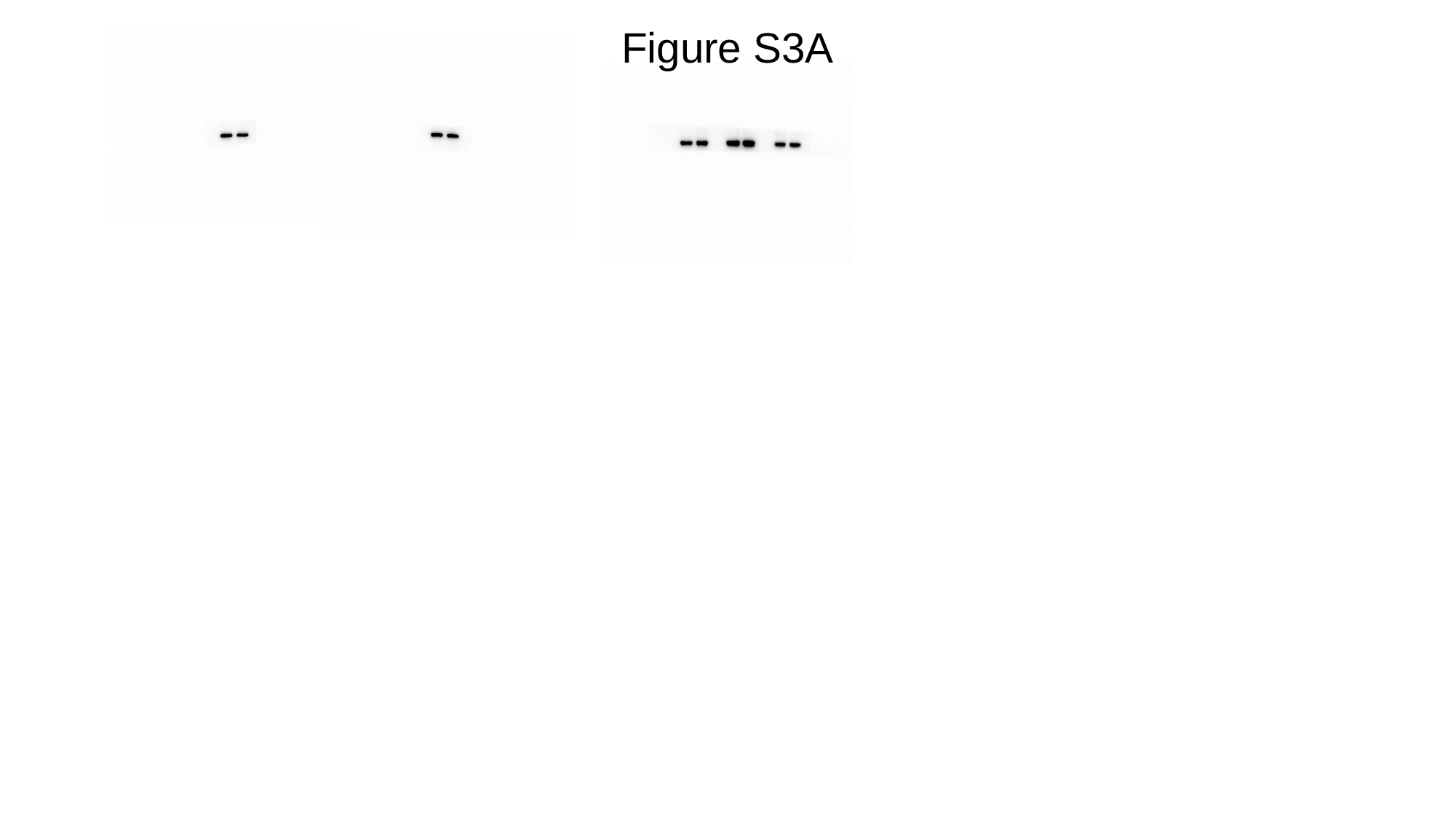

Figure S3A

## Slide 4
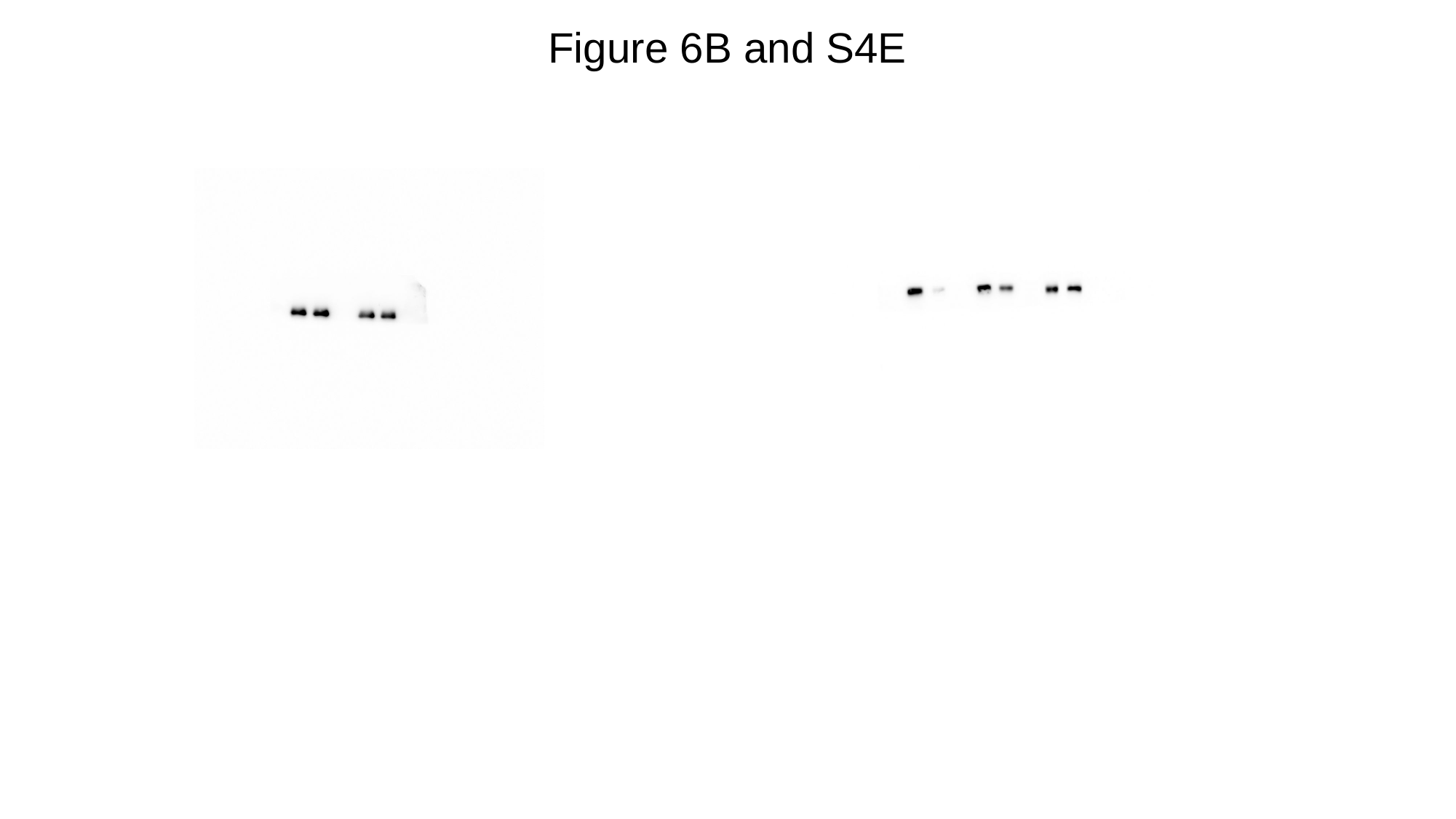

Figure 6B and S4E

Supplement: Supplementary file 2 — Raw Data [file 12276_2025_1540_MOESM2_ESM.zip › 新建文件夹/Raw-gel.pptx]
